# A New Hypoglycemic Prenylated Indole Alkaloid *N*-Oxide from Endophytic Fungus *Pallidocercospora crystalline*

**DOI:** 10.3390/ijms24108767

**Published:** 2023-05-15

**Authors:** Shuo Gao, Xiao Lin, Yeqin Shi, Hu Zhou, Xiao Zheng, Mingyu Li, Ting Lin

**Affiliations:** 1 Fujian Provincial Key Laboratory of Innovative Drug Target Research, School of Pharmaceutical Sciences, Xiamen University, Xiamen 361102, China; gaoshuoly@stu.xmu.edu.cn (S.G.); 32320191153416@stu.xmu.edu.cn (Y.S.); huzhou@xmu.edu.cn (H.Z.); zxiao@xmu.edu.cn (X.Z.); 2 Department of Basic Medical Sciences, School of Medicine, Xiamen University, Xiamen 361102, China; bvfox@163.com

**Keywords:** prenylated indole alkaloid, *N*-oxides, endophytic fungus, hypoglycemic activity, zebrafish

## Abstract

A new prenylated indole alkaloid—Penicimutamide C *N*-oxide (**1**), a new alkaloid penicimutamine A (**2**), along with six known alkaloids were isolated from an endophytic fungus *Pallidocercospora crystallina*. A simple and accurate method was used to determine the N-O bond in the *N*-oxide group of **1**. By using a β-cell ablation diabetic zebrafish model, compounds **1**, **3**, **5**, **6** and **8** showed significantly hypoglycemic activities under the concentration of 10 μM. Further studies revealed that compounds **1** and **8** lowered the glucose level through promoting glucose uptake in zebrafish. In addition, all eight compounds showed no acute toxicity, teratogenicity, nor vascular toxicity in zebrafish under the concentrations range from 2.5 μΜ to 40 μM. Importantly, these results provide new lead compounds for the development of antidiabetes strategies.

## 1. Introduction

*N-*oxides are very common organic molecules. Applications of *N-*oxides in chemistry and biology have attracted increasing interest, especially their pharmaceutical applications. Minoxidil is a pyrimidine *N-*oxide drug currently used for the treatment of any form of alopecia. Chlordiazepoxide is another *N-*oxide drug which has been used in the treatment of anxiety disorders [1]. More and more active *N-*oxides have been found in natural products [2,3,4,5]. The identification of the structure of *N-*oxides in natural products is very important, too. X-ray single crystal diffraction is the most accurate and intuitive method to determine the structure of *N-*oxides. However, many natural products cannot form single crystals. In cases like this, most of the structures were determined by the deshielding effects of the C and H signals adjacent to the N-O bond in the ^13^C-NMR and ^1^H-NMR spectra and the unsaturation from the mass spectrum. However, if there was an N-O inner ring instead of an N-O coordination bond in the structure, a similar deshielding effect in NMR data and the same unsaturation still existed [6]. In addition, in some MS/MS data of *N-*oxides, fragments of [M-16] could be found to prove the structures of *N-*oxides. However, the N-O coordination bond is stable in some *N-*oxides [7], and not all *N-*oxides have fragments of [M-16] in their MS/MS data. Therefore, these methods alone cannot accurately identify the structures of *N-*oxides.

**Figure 1:**
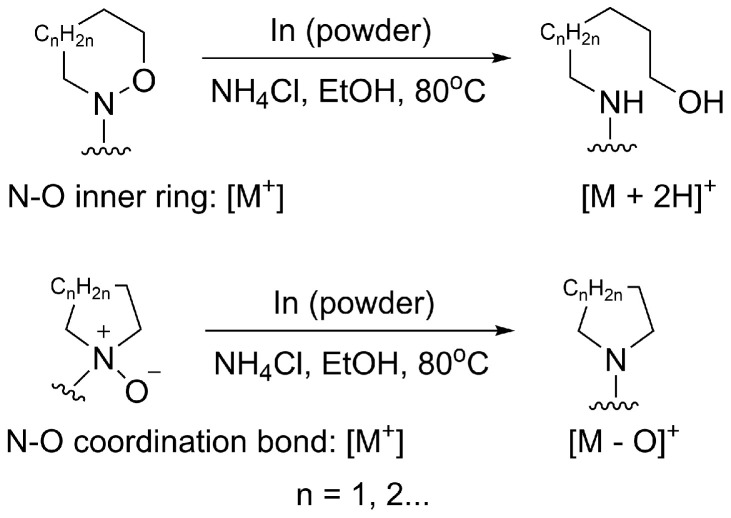
Indium-mediated reduction of N-O bonds.

In this study, we used a simple and accurate method to determine the structure of *N-*oxides. Indium powder was used as a mild reductant under weakly acidic conditions to break the N-O bond [8]. As shown in Figure 1, different products with different molecular weights will be obtained using this method. Compounds with N-O inner rings will undergo indium-mediated reduction reactions that break the N-O rings and add two hydrogens to the compounds [8,9]. Therefore, we can obtain the M + 2H reaction product and find the corresponding molecule ion peaks from the HR-MS data. As for *N*-oxides, via the subsequent In-mediated reduction with the lost oxygen molecule weight observed in HR-MS data, the N-O coordination bonds can be recognized directly.

**Figure 2:**
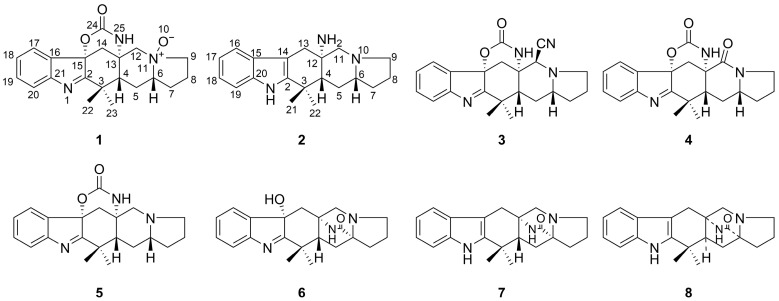
Structures of compounds **1**–**8**.

Prenylated indole alkaloids are a broad class of fungal secondary metabolites [10]. They are hybrid natural products containing indole/indoline and isoprenoid moieties [11]. Many of them are composed of _L_-tryptophan, a second cyclic amino acid residue, and one or two isoprene units [12]. They have often been isolated from the fungal genera *Penicillium* and *Aspergillus* and exhibit various biological activities such as antitumor, anthelmintic, calmodulin inhibitory and antibacterial effects [13]. *N-*oxides in natural prenylated indole alkaloids are not very common. In our study, penicimutamide C *N*-oxide (**1**), a new prenylated indole alkaloid *N-*oxide, was isolated from an endophytic fungus, *Pallidocercospora crystallina*, of the plant *Ajuga decumbens*. The chemical structure (Figure 2) of **1** was elucidated through the analysis of spectroscopic data (1D and 2D NMR), HR-ESI-MS data and electronic circular dichroism (ECD) calculations. The *N-*oxide group in **1** was confirmed using the method, which combined a reduction reaction and MS data analysis and the computational quantum chemistry method. Furthermore, another new prenylated indole alkaloid penicimutamine A (**2**) was characterized along with six known prenylated indole alkaloids: aspeverin (**3**) [14,15], penicimutamide A (**4**) [15], penicimutamide C (**5**) [15], penicimutamide D (**6**) [10], penicimutamide E (**7**) [10] and (±)-premalbrancheamide (**8**) [10], from the strain *Pallidocercospora crystallina*. Their structures were determined via NMR data (1D and 2D NMR) analysis and comparisons with data in the literature (Tables S1–S4).

## 2. Results and Discussion

### 2.1. Reduction Reactions of Known N-Oxide Compounds

Seven known natural *N-*oxides, emeheterone, alstoyunine E, seneciphylline *N-*oxide, isotetrandrine *N*-2′-oxide, paxiphylline D, nicotine 1′-*N-*oxide, oxymatrine and a synthetic *N-*oxide clozapine *N-*oxide, were used to verify the reliability of this method, and a completely positive conclusion was drawn. The loss of the oxygen molecular ion peaks in all eight compounds was observed from the HR-ESI-MS data after the In-mediated reductions.

The eight *N-*oxides were subjected to reductions via indium under a mixed ethanol/saturated aqueous NH_4_Cl solution and heated to 80 °C for 4 h. The resulting mixture was quenched with Na_2_CO_3_ and extracted three times with ethyl acetate. The combined organic layers were dried with nitrogen and then analyzed via HR-ESI-MS. From the HR-ESI-MS data, we could find the molecular ion peaks (Table 1) which were 16 less than the mass of the corresponding unreduced *N*-oxides (the MS data are shown in the Supplementary Materials Figures S1–S8). These results indicated that the N-O coordination bond could be reduced and broken using indium powder. The method could be used to determine whether natural products contain *N-*oxide groups.

### 2.2. Structural Elucidation

Penicimutamide C *N*-oxide (**1**) was obtained as white amorphous powder. The molecular formula of **1** was established as C_21_H_25_O_3_N_3_ (eleven degrees of unsaturation) by its (+)-HR-ESI-MS at *m*/*z* 390.1783 [M + Na]^+^ (calcd. for C_21_H_25_O_3_N_3_Na, 390.1794) and its (−)-HR-ESI-MS at *m*/*z* 402.1592 [M + Cl]^−^ (calcd. for C_21_H_25_O_3_N_3_Cl, 402.1584). The ^1^H NMR data (Table 2) of **1** showed 24 proton signals, including 4 phenyl group protons at δ_H_ 7.33 (1H, td, *J* = 7.4, 0.9 Hz), δ_H_ 7.47 (1H, td, *J* = 7.6, 1.1 Hz), δ_H_ 7.53 (1H, br d, *J* = 7.8 Hz) and δ_H_ 7.54 (1H, br d, *J* = 7.9 Hz), and 2 upfield methyls at δ_H_ 1.31 (3H, s) and δ_H_ 1.50 (3H, s). The ^13^C NMR and DEPT spectra of **1** exhibited 21 carbon resonances, including 2 methyls, 6 *sp*^3^ methylenes, 2 *sp*^3^ methines, 3 *sp*^3^ quaternary carbons, 4 *sp*^2^ methines and 4 *sp*^2^ quaternary carbons. In combination with ^1^H NMR data and six olefinic carbons at δ_C_ 121.8, δ_C_ 124.4, δ_C_ 128.2, δ_C_ 132.1, δ_C_ 137.1 and δ_C_ 153.8, **1** was found to possess a disubstituted phenyl group. Because of the eleven degrees of unsaturation, we speculated **1** was a polycyclic structure.

**Figure 3:**
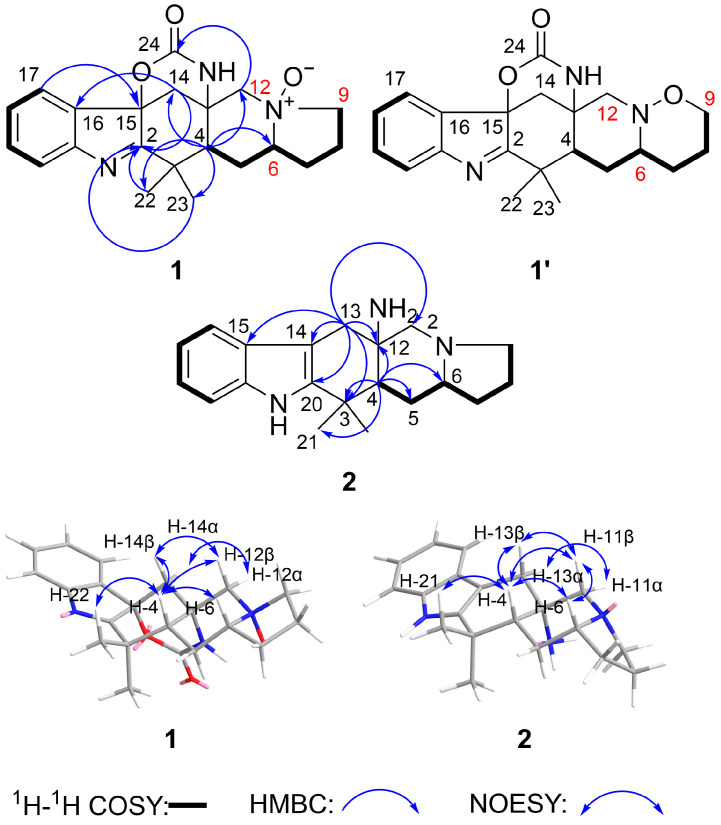
Key 1H-1H COSY, HMBC and key NOESY correlations for **1** and **2**.

The 2D NMR spectra were important to confirm the structure of **1**. From the ^1^H-^1^H COSY spectrum correlations of H-17/H-18/H-19/H-20 and H-4/H-5/H-6/H-7/H-8/H-9, two partial structure units could be established. The HMBC spectrum showed the connections of these substructures between H-17/C-15; H-14/C-2, C-16; H-22/C-2; H-23/C-2; H-12/C-24; H-4/C-3, C-6, C-12, C-13, C-14, C-22, C-23 (Figure 3). From HR-ESI-MS data, we found **1** had an *m*/*z* that was 16 higher than **5**. So, we believed **1** had one oxygen atom more than **5**. Comparing the ^13^C NMR of **1** and **5**, we found the chemical shifts at C-6 (δ_C_ 75.9), C-9 (δ_C_ 69.0) and C-12 (δ_C_ 68.9) of **1** were quite different from C-6 (δ_C_ 65.6), C-9 (δ_C_ 54.2) and C-11 (δ_C_ 62.9) of **5**. So, a N-O bond must exist at N-11. Additionally, the exact structure of N-O bond could not be confirmed by the weak correlation signal between H-12α/C-9. Meanwhile, from the (+)-HR-ESI-MS/MS data of compound **1** and **5** (Figures S40 and S41), we found **1** and **5** may have similar MS fragmentation patterns (Schemes S1 and S2), and there is no fragment of [M-16] in MS/MS data of **1**. So, there were two different possible structures: *N-*oxide (**1** in Figure 3) and a compound containing an N-O bond six-ring system (**1′** in Figure 3).

**Scheme 1:**
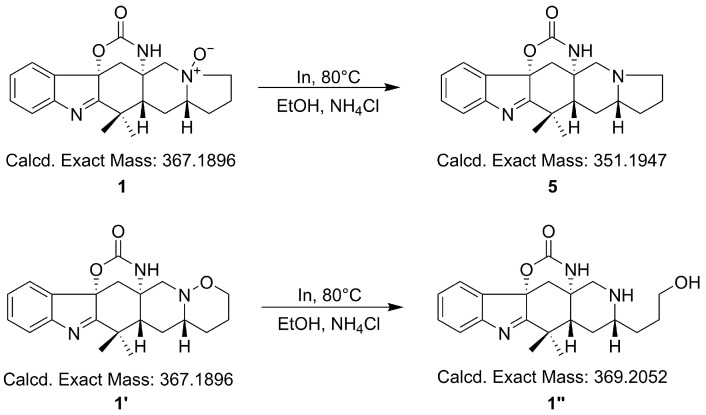
Possible reaction process of compound **1**.

In order to confirm which was the precise N-O bond at N-11, we used the aforementioned method to perform a reduction reaction on compound **1** by using indium powder under weak acid conditions. **1** and **1′** could be reduced to different compounds and had different molecular weights (Scheme 1).

**Figure 4:**
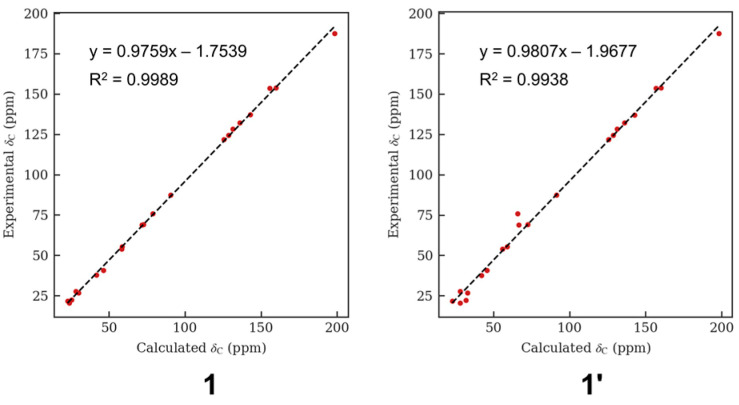
Regression analysis of experimental versus calculated ^13^C NMR chemical shifts of **1** and **1′**.

After analyzing the HR-ESI-MS data of the reaction product, we could find *m*/*z* 352.2018, which demonstrated *N*-oxide was the right N-O bond in **1** (Figure S42). For further verification, the computational quantum chemistry method was used to compute the ^13^C-NMR chemical shifts of **1** and **1′**. They were calculated at the mPW1PW91/6-311G (2d,p) level in the gas phase via Gaussian 09. The computational ^13^C-NMR data were finally obtained using the linear regression analysis method. **1** agreed well with the experimental one with the correlation coefficient (R^2^) of 0.9989 compared to that of **1′** (0.9938) (Figure 4).

The relative stereochemistry of **1** was determined by analyzing the NOESY spectrum (Figure 3). In the NOESY spectrum, H-4 correlated with H-6, H-22, H-12β and H-14β; H-12β correlated with H-14β; this suggested that these protons were situated on the same face and were β-oriented. H-12α exhibited correlations with H-14α, which suggested these two protons were α-oriented. Because H-4, H-12β and H-14β were correlated with each other and at the same face, the carbamate ring (C13-N25-C24-O-C15) should have been at the opposite face which was α-oriented.

**Figure 5:**
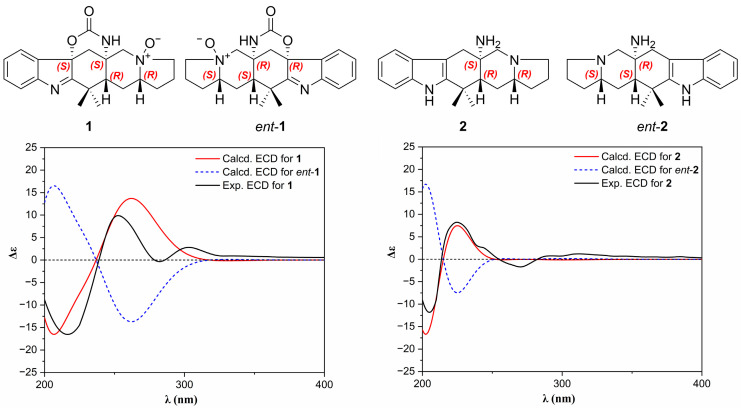
Experimental and calculated ECD spectra of **1** and **2** in methanol.

The absolute stereochemistry of **1** was elucidated via a circular dichroism (CD) experiment (Figure 5). The electronic CD calculations for **1** and its enantiomer (*ent*-**1**) were performed. The calculated ECD of **1** agreed with the measured ECD data. So, the absolute stereochemistry of **1** was 4R, 6R, 13S, 15S.

Penicimutamine A (**2**) was obtained as yellow amorphous powder. The molecular formula of **2** was established as C_20_H_27_N_3_ by its (+)-HR-ESI-MS at *m*/*z* 310.2275 [M + H]^+^ (calcd. for C_20_H_28_N_3_, 310.2283) and its (−)-HR-ESI-MS at *m*/*z* 344.1901 [M + Cl]^−^ (calcd. for C_20_H_27_N_3_Cl, 344.1894), indicating nine degrees of unsaturation. The ^1^H and ^13^C NMR spectra of **2** (Table 2) were similar to those of penicimutamide E (**7**). The ^1^H NMR spectrum at δ_H_ 7.08 (1H, td, *J* = 7.2, 1.1 Hz), δ_H_ 6.99 (1H, td, *J* = 7.9, 0.8 Hz), δ_H_ 7.40 (1H, br d, *J* = 7.8 Hz) and δ_H_ 7.31 (1H, br d, *J* = 8.0 Hz) revealed a disubstituted phenyl group in the indole structure. Comparing the MS data and the molecular formulas between **2** and **7**, it was easy to find that **7** had more oxygen and carbon than **2**. Additionally, the degree of unsaturation of **2** was two less than that of **7**. Combined with the ^1^H and ^13^C NMR data, we speculated that the lactam ring between C-6 and C-12 broke and the carbonyl on C-6 disappeared. The HSQC correlations from proton δ_H_ 2.13 (H-6) to δ_C_ 66.0 (C-6) confirmed the speculation. The HMBC spectrum showed correlations from δ_H_ 1.97 (H-4) to δ_C_ 24.2 (C-21)/δ_C_ 28.1 (C-5)/δ_C_ 34.7 (C-3)/δ_C_ 56.5 (C-12)/δ_C_ 66.0 (C-6), δ_H_ 2.86 (H-13) to δ_C_ 49.8 (C-4)/δ_C_ 56.5 (C-12)/δ_C_ 63.3 (C-11)/δ_C_ 102.7 (C-14)/δ_C_ 128.3 (C-15)/δ_C_ 141.5 (C-2), which deduced the planar structure of **2** (Figure 3).

The relative stereochemistry of **2** was determined by analyzing the NOESY spectrum (Figure 3). In the NOESY spectrum, H-4 correlated with H-6, H-21, H-11β and H-13β; H-11β correlated with H-13β and H-6. These correlations suggested that these protons were situated on the same face and were β-oriented. On the contrary, the NH_2_ group on C-12 should be α-oriented. H-11α exhibited correlations with H-13α, which suggested these two protons were α-oriented. The absolute stereochemistry of **2** was elucidated via a circular dichroism (CD) experiment (Figure 5). The electronic CD calculations for **2** and its enantiomer (*ent*-**2**) were performed. The calculated ECD of **2** agreed with the measured ECD data. So, the absolute stereochemistry of **2** was 4R, 6R, 12S.

### 2.3. Hypoglycemic Activities of These Compounds

The zebrafish (*Danio rerio*) is a valuable model organism with applications in many subjects, especially in biomedical research [16,17]. It is also an ideal model for studying diabetes and antidiabetes drug screening [18,19]. Its glucose metabolism and the pathways of reactive metabolite formation are very similar to those of humans [20,21]. Simultaneously, the zebrafish embryonic toxicity model can be used to evaluate the toxicity of bioactivity compounds [22]. We used the zebrafish diabetes models to detect the hypoglycemic activity of the eight compounds isolated from the fungi to find new lead compounds for the development of antidiabetes strategies.

**Figure 6:**
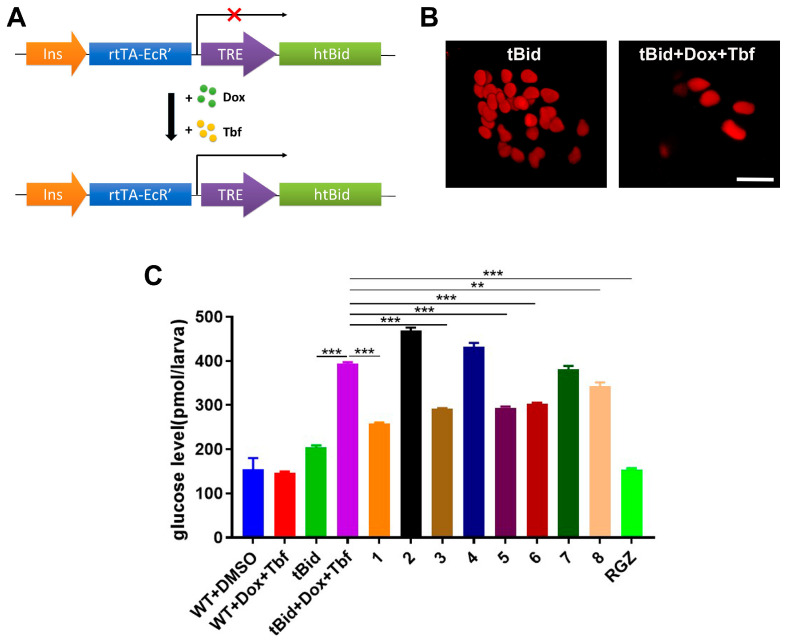
Compounds decreased the glucose level in a diabetic zebrafish model: (**A**) Schematic representation of zebrafish diabetic model *Tg(−1.2ins:htBid^TE-ON^; LR)*. Zebrafish insulin promoter was employed to drive the proapoptotic gene human truncated Bid (htBid) under the control of a TRE-based system. After incubation with doxycycline (Dox) and tebufenozide (Tbf), the htBid will express and cause β-cell apoptosis. The htBid will not express without incubation of Dox and Tbf (red cross). (**B**) Representative confocal images of the β-cell number from *Tg(−1.2ins:htBidTE-ON; LR)*; *Tg(−1.2ins:H2BmCherry)* larvae treated with (tBid + Dox + Tbf) or without (tBid) doxycycline and tebufenozide β cells were indicated by the fluorescence of mCherry. Scale bars indicate 10 μm. (**C**) Total free glucose level of 6 dpf *Tg(−1.2ins:htBid^TE-ON^; LR)* treated with or without compounds. Total of 10 μM of each compound was added at 5 dpf after the β-cell ablation, and lasted for 24 h; then, their glucose levels were measured. WT: wild type. RGZ: rosiglitazone, which was used as positive control. The values shown are means ± SEM from three independent experiments. ** *p* < 0.01, and *** *p* < 0.001 via one-way ANOVA.

To survey the hypoglycemic activities of these compounds, a zebrafish β-cell ablation model, *Tg(−1.2ins:htBid^TE-ON^; LR)*, was applied [23]. The truncated human Bid protein (htBid) was derived by an insulin promoter and controlled under the tetracycline- and ecdysone-inducible system (Figure 6A). After induction with doxycycline (Dox) and tebufenozide (Tbf), the proapoptotic tBid expression in β cells resulted in the apoptosis of β cells, which were labeled by the transgenic line *Tg(−1.2ins:H2Bmcherry)* (Figure 6B). We then processed the model for a bioactive test via co-incubation with these eight compounds (10 μM) for 24 h. As shown in Figure 6C, the free glucose level was significantly increased after the β cells’ ablation compared with the non-induced transgenic zebrafish (tBid + Dox + Tbf vs. tBid, 394 ± 2.9 pmol/larva vs. 204 ± 3.9 pmol/larva, *p* < 0.001), suggesting that the β cells’ ablation caused zebrafish hyperglycemia. Interestingly, the glucose levels of hyperglycemia zebrafish treated with compounds **1**, **3**, **5**, **6** and **8** were 258 ± 2.2 pmol/larva, 291± 2.3 pmol/larva, 294 ± 2.1 pmol/larva, 303 ± 2.0 pmol/larva and 342.5 ± 8.7 pmol/larva, respectively, which significantly reduced the glucose levels (vs. tBid+Dox+Tbf, 394 ± 2.9 pmol/larva, *p* < 0.001 or *p* < 0.01), which revealed that these compounds have hypoglycemic activities. Moreover, the glucose level in the compound **1** group was lower than **5** (258 ± 2.2 pmol/larva vs. 294 ± 2.1 pmol/larva)**,** which indicated that **1** had higher hypoglycemic activity than **5**.

**Figure 7:**
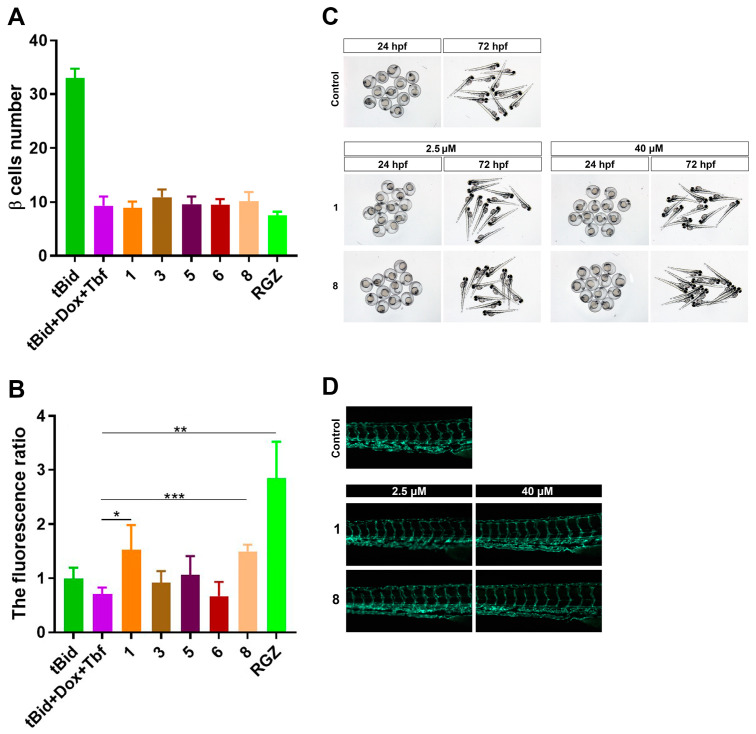
Compounds increased glucose uptake without any apparent toxicity: (**A**) The β-cell number of each compound-treated double transgenic zebrafish *Tg(−1.2ins:htBid^TE-ON^; LR) Tg(−1.2ins:H2Bmcherry)*. Total of 10 μM of each compound was added at 5 dpf after the β-cell ablation for 24 h. (**B**) The glucose uptake indicated by 2-NBDG fluorescence. The larvae of each group were induced with doxycycline and tebufenozide except the group of tBid in 3 dpf for 48 h. Total of 10 μM of each compound was added at 5 dpf after the β-cell ablation for 24 h. Then, 2-NBDG was incubated for 3 h in dark. RGZ: rosiglitazone. The values shown are means ± SEM from three independent experiments. * *p* < 0.05, ** *p* < 0.01 and *** *p* < 0.001 via one-way ANOVA. (**C**) Morphology of 24 hpf embryos or 72 hpf zebrafish larvae treated with compounds. (**D**) Blood vessel morphology of 6 dpf zebrafish larvae *Tg(flil:eGFP)* treated with compounds.

To further investigate the mechanism of the hypoglycemic activity of these compounds, we tested them through two mechanisms, by increasing β-cell regeneration and by the promotion of glucose uptake. Firstly, we counted the number of β cells after the compounds’ treatment using double transgenic zebrafish *Tg(−1.2ins:htBid^TE-ON^; LR)*; *Tg(−1.2ins:H2Bmcherry)*. Compared with the β-cell ablation group (tBid + Dox + Tbf), no differences in β-cell number were observed after they were treated with these compounds (Figure 7A). With the negative results from β-cell regeneration, we then turned to the glucose uptake mechanism. After the β-cell ablation, the 2-NBDG, a glucose fluorescence analog, was added in the zebrafish culture medium for indication of glucose uptake. Remarkably, we found that compounds **1** and **8** significantly increased the glucose uptake indicated by the 2-NBDG fluorescence (**1** vs. tBid + Dox + Tbf, 1.53 ± 0.45 vs. 0.70 ± 0.12, *p* < 0.05; **8** vs. tBid + Dox + Tbf, 1.50 ± 0.12 vs. 0.70 ± 0.12, *p* < 0.001) (Figure 7B). However, compound **5** did not change the glucose uptake (**5** vs. tBid+Dox+Tbf, 1.06 ± 0.35 vs. 0.70 ± 0.12, *p* = 0.125) (Figure 7B). Taken together, these data suggested that compounds **1** and **8** reduce the glucose level by the promotion of glucose uptake instead of the induction of β-cell regeneration.

Moreover, we evaluated the toxicity of the eight compounds (ranging from 2.5 μΜ to 40 μM) using zebrafish larvae as a model. We did not find any morphological change, malformation, mortality or developmental delay from zebrafish treated with compounds for 24 hpf or 72 hpf (Figure 7C and Figure S43). We also tested the vascular toxicity using transgenic line *Tg(fli1:eGFP)*, which labeled the vascular endothelial cells with eGFP. As shown in Figure 7D and Figure S44, the vascular morphology of 6 dpf zebrafish larvae appeared normal without any apparent change compared with the control group. These data suggested that these compounds have no apparent acute toxicity, teratogenicity or vascular toxicity to zebrafish. Comparing the structures of these eight compounds and their hypoglycemic activities, we found penicimutamine A (**2**) has no lactam ring, neither at C-6 to C-12 nor at C-12 to C-14. Penicimutamine A (**2**) showed no hypoglycemic activity, indicating that the lactam ring may be a necessary active group. When the carbamate ring is at C-12 to C-14, the carbonyl group at C-11 (compound **4**) can weaken the hypoglycemic activity. When the lactam ring is at C-6 to C-12, the hydroxy group at C-14 (compound **6**) can improve the hypoglycemic activity. Penicimutamide E (**7**) and (±)-premalbrancheamide (**8**) have the same planar constructions, but their absolute configurations are not the same. When the carbamate ring at C-6 to C-12 and the proton at C-4 were situated on the same face (compound **8**), the hypoglycemic activity was improved. We found that the hypoglycemic activity between compound **1** and **5** was significantly increased. So, we considered that the N-O bond in the *N*-oxide group may be associated with the higher hypoglycemic activity in **1**.

### 2.4. Plausible Biosynthetic Pathway

**Scheme 2:**
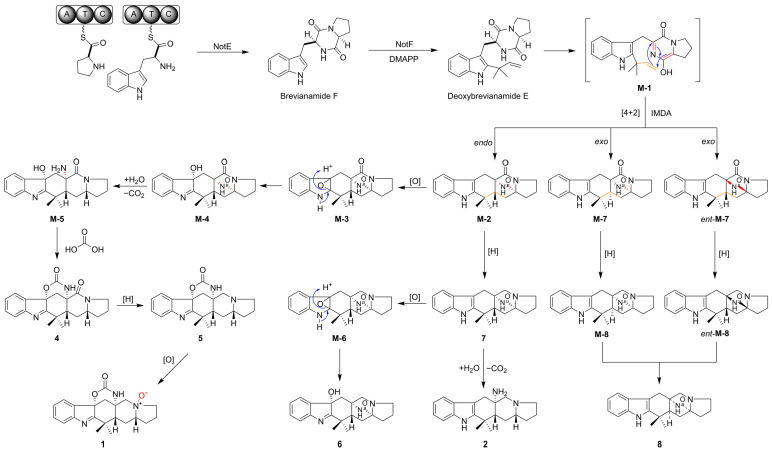
A plausible biosynthetic pathway for **1**–**8**. The blue arrow indicates the direction of electron movement. The red and orange bonds in M-1, M-2, M-7 and ent-M-7 indicate the change in position of the bonds before and after the IMDA reaction. The red bonds in M-4, M-5 and 1 indicate the bonds which are involved in the reaction.

We proposed a biosynthetic pathway for **1**–**8**, as shown in Scheme 2. Firstly, _L_-tryptophan and _L_-proline would be condensed with the catalysis of NotE (a presumed bimodular NRPS) to produce brevianamide F. With the function of NotF (a deoxybrevianamide E synthase), a DMAPP would be transferred to brevianamide F to afford deoxybrevianamide E [12]. Then, deoxybrevianamide E would produce intermediate **M-1** and then produce **M-2** via an intramolecular *endo* Diels–Alder (IMDA) [4 + 2] cyclization and produce **M-7** and *ent*-**M-7** via intramolecular *exo* Diels–Alder (IMDA) [4 + 2] cyclization [15,24]. **M-7** and *ent*-**M-7** would produce **M-8** and *ent*-**M-8** via a reduction reaction, respectively. Compound **8** is a mixture of **M-8** and *ent*-**M-8**. **M-2** would produce **M-3** via epoxidation and then the ring-opening of the epoxide to obtain **M-4**. Through a hydrolysis reaction and decarboxylation reaction, **M-4** would produce **M-5**. **M-5** would produce **4** by reacting with a molecule of carbonic acid. **4** would produce **5** via a reductase. **1** could be obtained from **5** through oxidation. In addition, **M-2** would produce **7** via a reduction reaction. **7** would produce **2** via a hydrolysis reaction and decarboxylation reaction. The epoxidation of **7** followed by the ring-opening of the epoxide would afford **6**.

## 3. Materials and Methods

### 3.1. General Experimental Procedures

The optical rotation values were measured with a Rudolph Autopol IV/IV-T automatic polarimeter (Rudolph Research Analytical, Hackettstown, NJ, USA). The IR spectra were obtained with a Nicolet iS50 FTIR Spectrometer (Thermo Fisher Scientific, Waltham, MA, USA). The UV spectra were measured with a Shimadzu UV-2600 UV-visible spectrophotometer (Shimadzu, Kyoto, Japan). The ECD spectra were collected using a Bio-Logic MOS 500 multifunctional hand spectrometer (Bio-Logic, Seyssinet-Pariset, France) in methanol. The NMR spectra were recorded on a Bruker Avance 600 III spectrometer (Bruker, Mannheim, Germany) with tetramethylsilane as the internal standard, using methanol-*d_4_* or DMSO-*d_6_* as the solvent. HRESIMS experiments were carried out on a Thermo Scientific Q Exactive Quadrupole- Orbitrap mass spectrometer (Thermo Fisher Scientific, Waltham, MA, USA). The column chromatography was performed with ODS (50 μm, YMC, Kyoto, Japan), Sephadex LH-20 (25–100 μm, Amersham Pharmacia Biotech, Stockholm, Sweden) and silica gel (200–300 mesh, Qingdao Marine Chemical Inc., Qingdao, China). Thin-layer chromatography (TLC) was carried out with precoated silica gel plates (GF_254_, Qingdao Marine Chemical Inc., Qingdao, China) and visualized under a UV lamp and by spraying with 10% H_2_SO_4_ solution in ethanol, followed by heating. The analytical HPLC was performed on a system comprising a pump (LC-20AT, Shimadzu, Kyoto, Japan), a dual 𝜆 absorbance detector (SPD-20A, Shimadzu, Kyoto, Japan), an ELS detector (ELSD-LT II, Shimadzu, Kyoto, Japan) and an RP-C18 column (5 μm, 4.6 × 250 mm, Welch Ultimate XB-C18, Welch Materials Inc., Shanghai, China). The semipreparative HPLC was performed on a system comprising a pump (LC-20AP, Shimadzu, Kyoto, Japan), a dual 𝜆 absorbance detector (SPD-20A, Shimadzu, Kyoto, Japan) and an RP-C18 column (5 μm, 10.0 × 250 mm, Welch Ultimate XB-C18, Shanghai, China).

### 3.2. Fungal Material

The endophytic fungus, strain A-S-6, was isolated from the current-year roots of the plant *Ajuga decumbens* collected from Fuzhou, Fujian, China. It was identified as *Pallidocercospora* genus according to a BLAST search result, which showed the internal transcribed spaces (ITS) sequence of the fungus was highly homologous (99% similarity) to the fungus *Pallidocercospora crystallina* (ID: KP896014.1). The ITS sequence was shown in Data S5.

### 3.3. Fermentation, Extraction, and Isolation

The A-S-6 strain was cultivated at 28 °C with 40 L of potato dextrose agar (PDA) media for 41 days. After the fermentation, the mycelium and media were chopped and soak extracted five times with a mixed solvent, EtOAc-MeOH (4:1, *v*/*v*), at room temperature for 12 h each time. The extract solution was filtered with gauze and evaporated under reduced pressure at 35 ± 2 °C to give a crude extract. The crude extract was liquid–liquid extracted 5 times with EtOAc-H_2_O (1:1). The EtOAc solution was then evaporated under reduced pressure at 35 ± 2 °C to give an EtOAc extract. The EtOAc extract was dissolved with MeOH and filtered, and then evaporated under reduced pressure at 35 ± 2 °C to give a MeOH extract (20.3 g).

The MeOH (20.3 g) extract was separated into 14 fractions (Fr. A-N) via ODS column chromatography with a gradient of MeOH-H_2_O (30:70, 50:50, 70:30, 100:0, *v*/*v*, 2 L for each step). Fr. D (60.0 mg) was separated using silica gel column chromatography with CH_2_Cl_2_-MeOH and purified via RP-HPLC with MeOH-H_2_O (50:50, *v*/*v*) to obtain compound **6** (1.0 mg). Fr. E (0.18 g) was subjected to the Sephadex LH-20 column with MeOH to yield six fractions (Fr. E1–E6). Fr. E1 (45.3 mg) was purified via RP-HPLC with MeOH-H_2_O (60:40, *v*/*v*) to afford compound **5** (25.5 mg). Compound **4** (17.3 mg) was obtained via recrystallization from Fr. F (0.47 g). The rest of Fr. F was separated using a Sephadex LH-20 column with MeOH to give ten fractions (Fr. F1–F10). Fr. F3 (123.3 mg) was separated into six fractions (Fr. F3A–F3F) via RP-HPLC with MeOH-H_2_O (55:45, *v*/*v*). Fr. F3A (3.8 mg) was purified via RP-HPLC with MeOH-H_2_O (30:70, *v*/*v*) to afford compound **1** (1.1 mg). Fr. H (0.31 g) was separated via RP-HPLC with MeOH-H_2_O (45:55, *v*/*v*) to afford six fractions (Fr. H1–H6). Fr. H5 (53.4 mg) was purified via RP-HPLC with MeOH-H_2_O (50:50, *v*/*v*) to afford compound **7** (2.6 mg). Fr. I (1.67 g) was subjected to a Sephadex LH-20 column with MeOH to obtain eight fractions (Fr. I1–I8). Fr. I4 (1.078 g) was subjected to repeating a Sephadex LH-20 column with MeOH to give four fractions (Fr. I4A–I4D). Fr. I4B (707.5 mg) was separated via RP-HPLC with MeOH-H_2_O (50:50, *v*/*v*) to obtain seven fractions (Fr. I4B1–I4B7). Fr. I4B5 (96.7 mg) was subjected to successive purifications via RP-HPLC with MeOH-H_2_O-formic acid (50:50:0.1, *v*/*v*) and MeOH-H_2_O-formic acid (25:75:0.1, *v*/*v*) to afford compound **2** (3.0 mg). Fr. I5 (166.0 mg) was chromatographed on normal-phase silica gel with cyclohexane-EtOAc (2:1, 1:1, 0:1, *v*/*v*) to give six fractions (Fr. I5A–I5F). Fr. I5B (8.6 mg) was purified via RP-HPLC with Acetonitrile-H_2_O (45:55, *v*/*v*) to afford compound **3** (4.9 mg). Fr. I5C (5.2 mg) was purified via RP-HPLC with Acetonitrile-H_2_O (45:55, *v*/*v*) to afford compound **8** (1.0 mg). The NMR spectra of compounds **1**–**8** were shown in Supplementary Materials (Figures S9–S33).

Penicimutamide C *N*-oxide (**1**): white amorphous powder; ${\left[\alpha \right]}_{D}^{25}$ −51 (*c* 0.05, MeOH); IR (KBr) *ν*_max_ 3334, 2917, 1647, 1456, 1019, 613, 503 cm^−1^ (Figure S38); UV (MeOH) *λ*_max_ (log *ε*) 203 (2.65), 219 (2.78) nm (Figure S36); ECD (MeOH) *λ*_max_ (Δ*ε*) 214 (−20.4), 257 (+11.0), 279 (−1.9), 301 (+3.7) nm; ^1^H (methanol-*d_4_*, 600 MHz) and ^13^C NMR (methanol-*d_4_*, 150 MHz) data, Table 2; (+)-HR-ESI-MS *m*/*z* 390.1783 [M + Na]^+^ (calcd. for C_21_H_25_O_3_N_3_Na, 390.1794) (Figure S34), (−)-HR-ESI-MS *m*/*z* 402.1592 [M + Cl]^−^ (calcd. for C_21_H_25_O_3_N_3_Cl, 402.1584).

Penicimutamine A (**2**): yellow amorphous powder; ${\left[\alpha \right]}_{D}^{25}$ −56 (*c* 0.05, MeOH); IR (KBr) *ν*_max_ 3284, 1636, 523, 500 cm^−1^ (Figure S39); UV (MeOH) *λ*_max_ (log *ε*) 209 (2.61), 223 (2.72) nm (Figure S37); ECD (MeOH) *λ*_max_ (Δ*ε*) 205 (−11.8), 225 (+8.2), 270 (−1.7) nm; ^1^H (methanol-*d_4_*, 600 MHz) and ^13^C NMR (methanol-*d_4_*, 150 MHz) data, Table 2; (+)-HR-ESI-MS *m*/*z* 310.2275 [M + H]^+^ (calcd. for C_20_H_28_N_3_, 310.2283) (Figure S35), (−)-HR-ESI-MS *m*/*z* 344.1901 [M + Cl]^−^ (calcd. for C_20_H_27_N_3_Cl, 344.1894).

### 3.4. Reduction Reaction Procedures

The compound (0.1 mg) was dissolved in a mixed solvent, EtOH-NH_4_Cl (2:1, *v*/*v*, 21 μL). Indium (1 mg) (Aladdin, Shanghai, China) was added to the reaction system. The reaction was heated to 80 °C for 4 h. The resulting mixture was quenched with Na_2_CO_3_ and extracted three times with ethyl acetate. The combined organic layers were dried with nitrogen, then analyzed via HR-ESI-MS.

### 3.5. Ablation of Beta Cells in Diabetic Zebrafish

Tebufenozide (3 μL, 50 mmol/L) and doxycline (3 μL, 100 mmol/L) were added into a 3.5 cm cell culture dish containing 6 mL 0.3 × Danieau’s buffer. Then, the dish was placed in a zebrafish incubator without light for 48 h to induce β cells of *Tg(−1.2ins:htBid^TE-ON^; LR)* in 3 dpf ablation.

### 3.6. Treatment of Diabetic Zebrafish with Compounds

Diabetic zebrafish that were ready to be used in experiments after β-cell ablation were rinsed with 0.3 × Danieau’s buffer to remove tebufenozide and doxycline. The larvae were placed into a 24-well plate at a density of 10 zebrafishes/well, in 2 mL of egg water. All compounds were made in 1000× stock solution (10 mmol/L) with DMSO and stored in light-protected Eppendorf tubes at −20 °C. For the treatment, each group was added accordingly with 2 mL of egg water treated with 2 μL of each of the compounds (10 mmol/L) to reach the final concentration of (10 μmol/L), and the control group was treated with the same amount of DMSO. The treatments lasted for 24 h in the zebrafish incubator. The group of tBid which was not induced with tebufenozide and doxycline was used as a control of normal zebrafish.

### 3.7. Total Glucose Level Test

After the compound treatment, a pool of 10 larvae was homogenized in 100 μL of sample buffer. The homogenate was spun at 10,000 rpm for 10 min. Free glucose in 10 μL of supernatant (equivalent of one larva) was determined according to the manufacturer’s instructions. Fluorescence (excitation, 520 nm; emission, 580–640 nm) was measured using a SpectraMax M5 Microplate Reader (Molecular Devices, San Jose, CA, USA). Each sample was measured for three pools.

### 3.8. 2-NBDG Test

After the compound treatment, zebrafish (6 dpf) were incubated in a culture medium containing 600 μM 2-NBDG (Apexbio, B6035, Houston, TX, USA) for 3 h. A pool of 5 larvae was homogenized in 100 μL of sample buffer. The homogenate was spun at 10,000 rpm for 10 min. The supernatant (30 μL) was placed into a 96-well plate to detect fluorescence (excitation, blue 475 nm; emission, 500–550 nm) using a SpectraMax M5 Microplate Reader (Molecular Devices). Each sample was measured for three pools [25].

### 3.9. Zebrafish Embryo Toxicity Test

Wild-type (AB strain) zebrafish (*Danio rerio*) and *Tg(flil:eGFP)* zebrafish were maintained in flow-through tanks with fish water (0.2% Instant Ocean salt in deionized water, pH 6.9–7.2, conductivity 480–510 μS/cm and hardness 53.7–71.6 mg/L CaCO_3_) with a photoperiod of 14/10 h light/dark. Embryos were obtained from spawning adults placed in groups of two males and one female in one spawning box overnight. Embryos were collected within 0.5 h of spawning and rinsed in fish water. Twelve zebrafish embryos per condition were exposed to compounds at the concentrations of 2.5 μM and 40 μM, and 0.1% DMSO served as the control. Zebrafish were selected from each group for visual observation and image acquisition every 24 h and 72 h.

### 3.10. β-Cell Regeneration

The compounds with 0.3× Danieau’s buffer were washed out after the compound treatment in 24 h. The larvae with 4% paraformaldehyde were fixed overnight at 4 °C and placed on a slide with aqua-mount (Richard-AllanScientific, Kalamazoo, MI, USA) with the right sides of the larvae facing up to expose the islets. The β-cell number was counted according to the RFP under a Zeiss AxioImager A1 microscope (Carl Zeiss, Jena, Germany) with 40× lens.

All procedures have been approved by the Xiamen University Institutional Animal Care and Use Committee (Protocol XMULAC20160089, 10 March 2016).

## 4. Conclusions

In summary, we have used an efficient method to determine the structure of *N*-oxides and identify a natural *N-*oxide, Penicimutamide C *N*-oxide (**1**), from the endophytic fungus *Pallidocercospora crystallina*. Penicimutamide C *N*-oxide showed hypoglycemic activity by promoting glucose uptake instead of the induction of β-cell regeneration. On the basis of these findings, a new molecular structure design has been promoted steadily for the treatment of diabetes in our laboratory, which will expand the chemical diversity for curing diabetes.

## Figures and Tables

**Table 1:** The HR-MS data of eight *N*-oxides.

Compounds	[M + H]^+^ (*m*/*z*) ^[a]^	[M-O + H]^+^ (*m*/*z*) ^[b]^
Emeheterone	323.1385	307.1437
Alstoyunine E	351.1694	335.1748
Seneciphylline *N*-oxide	350.1588	334.1644
Isotetrandrine *N*-2′-oxide	639.3051	623.2742
Paxiphylline D	384.2161	368.2213
Nicotine 1′-*N*-oxide	179.1176	163.1227
Oxymatrine	265.1904	249.1958
Clozapine *N*-oxide	343.1313	327.1365

^[a]^ Molecular ion peak of the compound before reduction reaction was observed from HR-MS data. ^[b]^ Molecular ion peak of the product after reduction reaction was observed from HR-MS data.

**Table 2:** The 600 MHz ^1^H NMR and 150 MHz ^13^C NMR data for **1** and **2** in methanol-*d_4_*.

No.	1		2	
	δ_C_	δ_H_ (*J* in Hz)	δ_C_	δ_H_ (*J* in Hz)
2	187.4		141.5	
3	40.7		34.7	
4	53.9	1.85 dd (12.3 3.1)	49.8	1.97 dd (13.0 3.8)
5	22.3	β 2.04 m	28.1	β 2.10 m
		α 2.29 m		α 1.63 m
6	75.9	3.41 dd (12.3 3.1)	66.0	2.13 m
7	26.7	2.08 m	31.4	β 1.61 m
				α 2.02 m
8	20.5	β 2.05 m	22.4	α 1.82 m
		α 2.24 m		β 1.89 m
9	69.0	β 3.38 m	54.4	α 2.28 q (8.9)
		α 3.52 td (9.5 1.4)		β 3.11 dd (8.6 2.1)
11			63.3	β 2.37 d (11.8)
				α 3.28 d (11.8)
12	68.9	β 3.40 d (12.2)	56.5	
		α 3.73 d (12.4)		
13	55.3		31.5	β 2.86 d (16.2)
				α 2.92 d (16.1)
14	37.7	β 1.73 d (13.7)	102.7	
		α 2.66 d (13.7)		
15	87.4		128.3	
16	137.1		118.5	7.40 br. d (7.8)
17	124.4	7.54 br. d (7.9)	119.8	6.99 td (7.9 0.8)
18	128.2	7.33 td (7.4 0.9)	122.5	7.08 td (7.2 1.1)
19	132.1	7.47 td (7.6 1.1)	111.9	7.31 br. d (8.0)
20	121.8	7.53 br. d (7.8)	138.7	
21	153.8		24.2	1.31 s
22	27.7	1.50 s	30.7	1.43 s
23	21.8	1.31 s		
24	153.6			

## Data Availability

The data are contained within the article or Supplementary Materials.

## Supplementary Materials

The following supporting information can be downloaded at: https://www.mdpi.com/article/10.3390/ijms24108767/s1.
