# Supplementary material for: A New Hypoglycemic Prenylated Indole Alkaloid N-Oxide from Endophytic Fungus Pallidocercospora crystalline
Source: Int J Mol Sci. 2023 May 15;24(10):8767. doi: 10.3390/ijms24108767 (PMC10217879; doi:10.3390/ijms24108767)
Supplement: Supplementary file 1 [file ijms-24-08767-s001.zip › ijms-2385717-supplementary.pdf]

Supplementary Material

A New Hypoglycemic Prenylated Indole Alkaloid *N*-Oxide from Endophytic Fungus *Pallidocercospora crystalline*

Shuo Gao [1,†], Xiao Lin [2,†], Yeqin Shi [1], Hu Zhou [1], Xiao Zheng [1], Mingyu Li [1,*] and Ting Lin [1,*]

[1] School of Pharmaceutical Sciences, Fujian Provincial Key Laboratory of Innovative Drug Target Research, Xiamen University, Xiamen, 361102, China

[2] Department of Basic Medical Sciences, School of Medicine, Xiamen University, Xiamen 361102, China

[*] Correspondence: limingyu@xmu.edu.cn (M. Li), linting@xmu.edu.cn (T. Lin).

[†] These authors contributed equally to this work.

Contents

Data S1. Positive HR-ESI-MS data of *N*-oxides and their reduction reaction product 2

Data S2. NMR, HR-ESI-MS, UV and IR Spectra 10

Data S3. Analysis of (+)-HR-ESI-MS/MS data of compound 1 and 5. 30

Data S4. Biological assay 33

Data S5. The BLAST search result of ITS sequence of fungus A-S-6. 35

Data S1. Positive HR-ESI-MS data of *N*-oxides and their reduction reaction products.

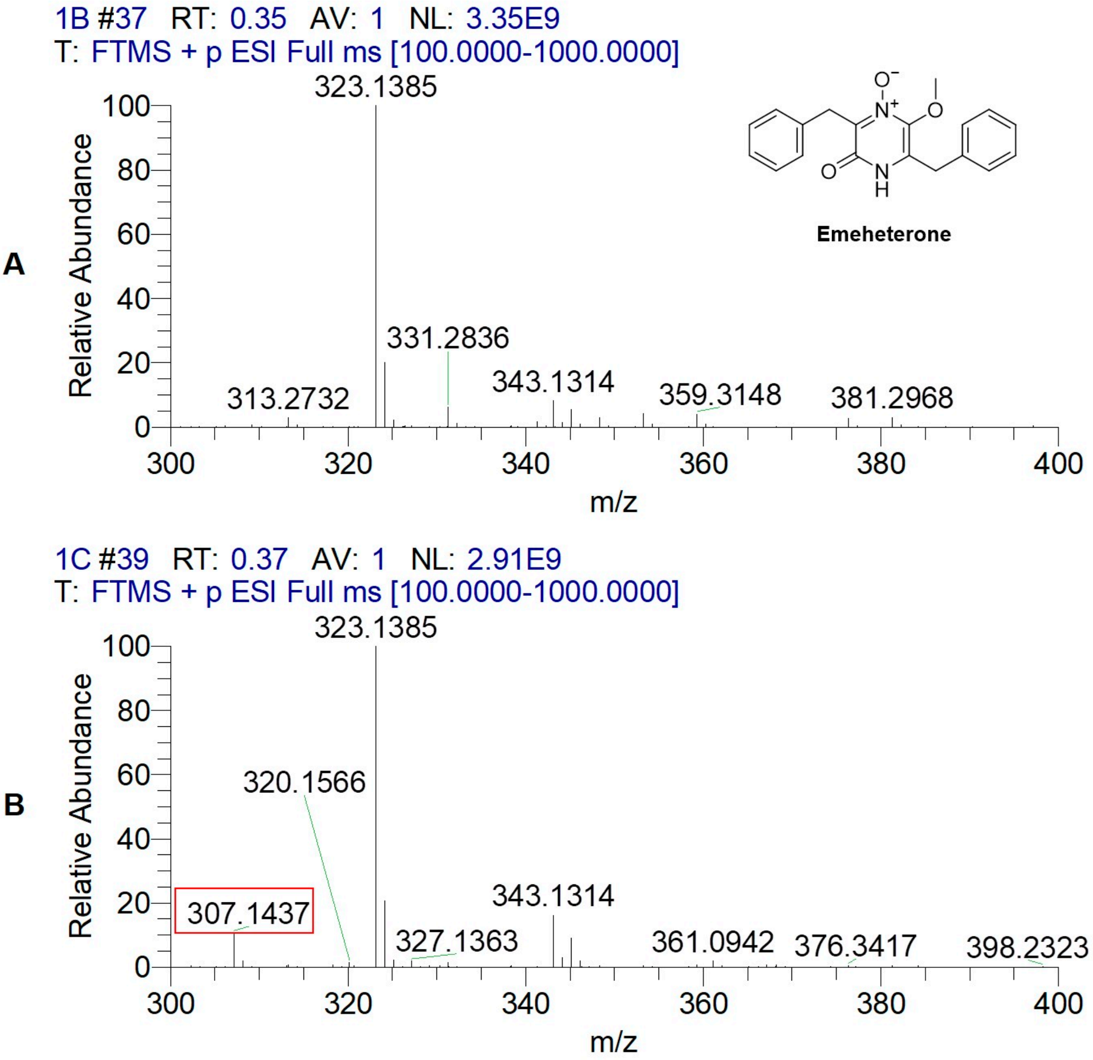

Figure S1. A: Positive MS of emeheterone; **B**: Positive MS of reduction reaction product of emeheterone (red box) by In.

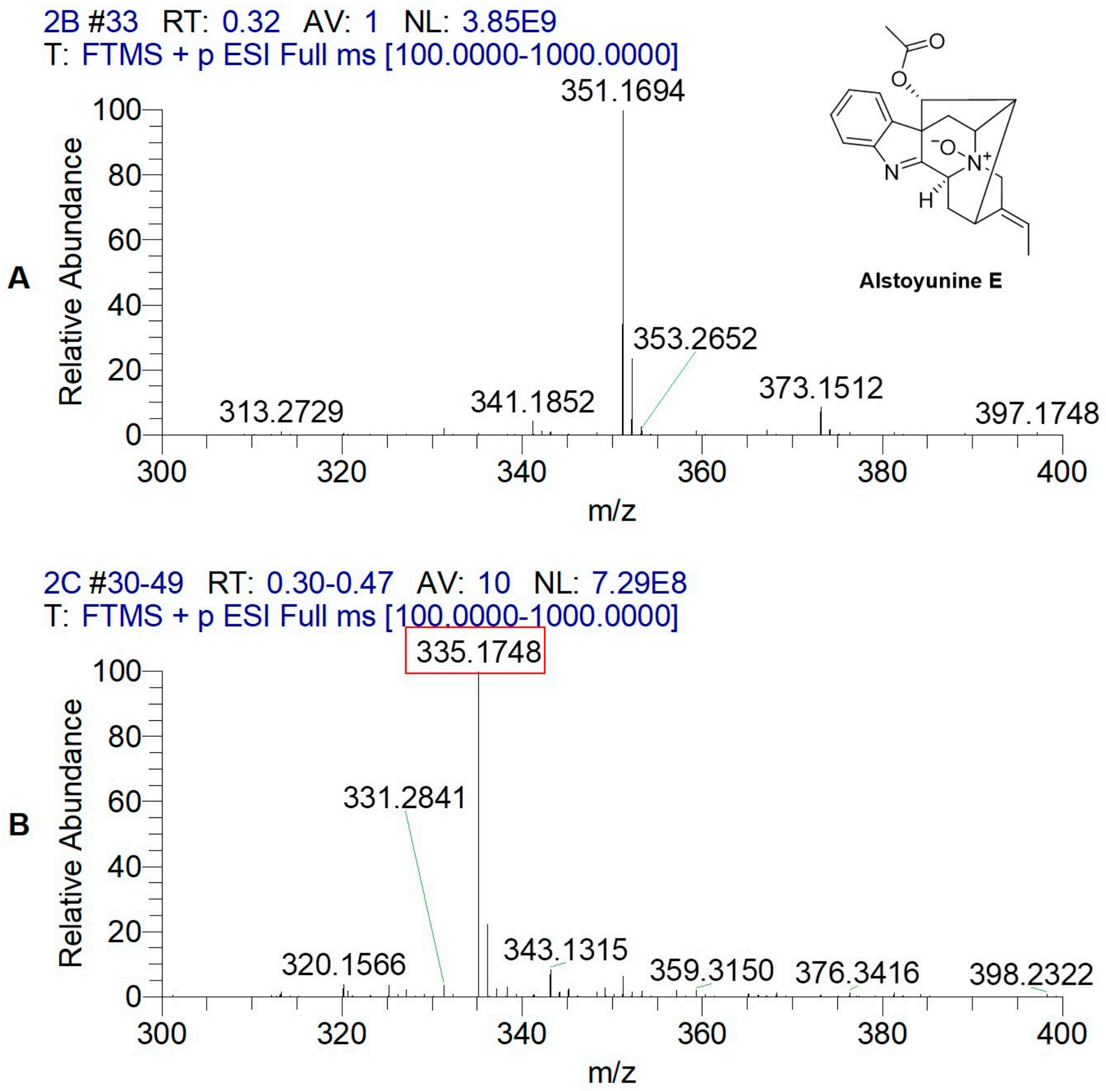

Figure S2. A: Positive MS of alstoyunine E; **B**: Positive MS of reduction reaction product of alstoyunine E (red box) by In.

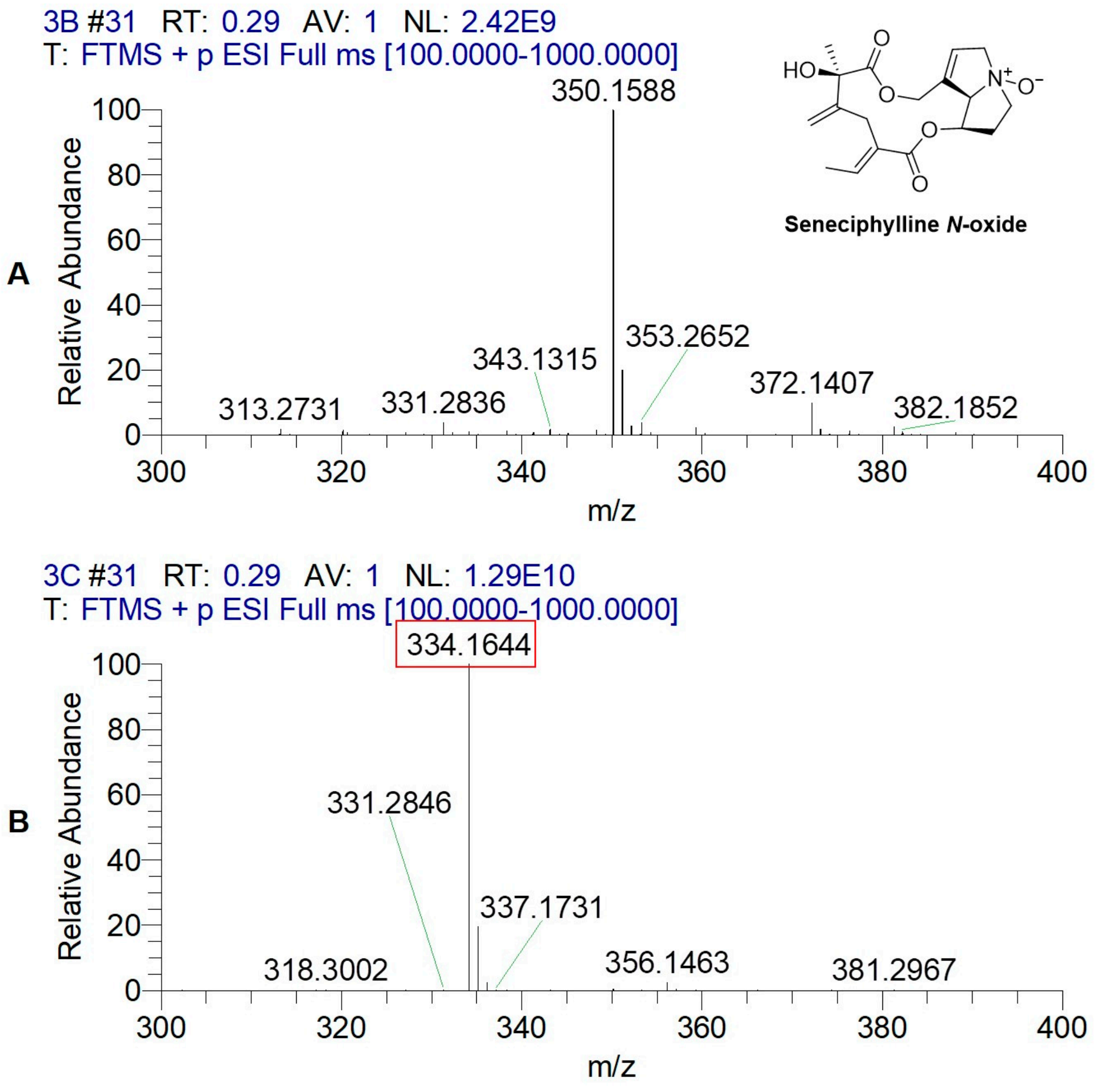

Figure S3. **A**: Positive MS of seneciphylline *N*-oxide; **B**: Positive MS of reduction reaction product of seneciphylline *N*-oxide (red box) by In.

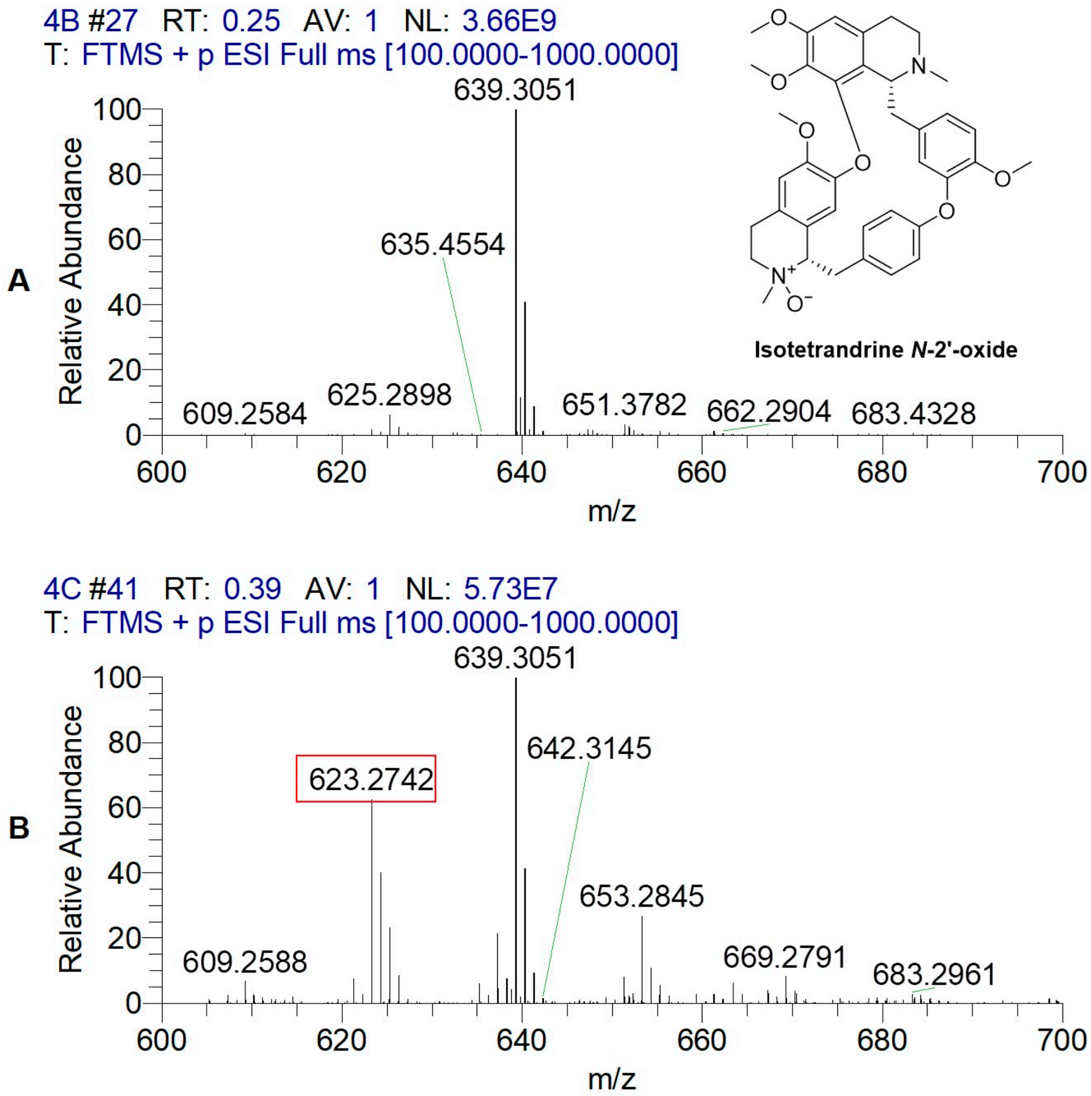

Figure S4. A: Positive MS of isotetrandrine *N*-2'-oxide; **B**: Positive MS of reduction reaction product of isotetrandrine *N*-2'-oxide (red box) by In.

A

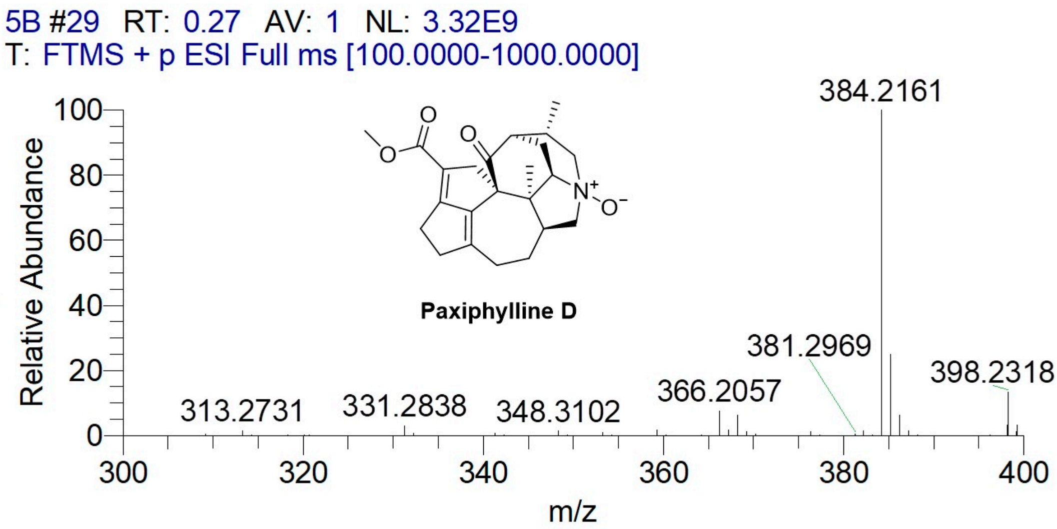

B

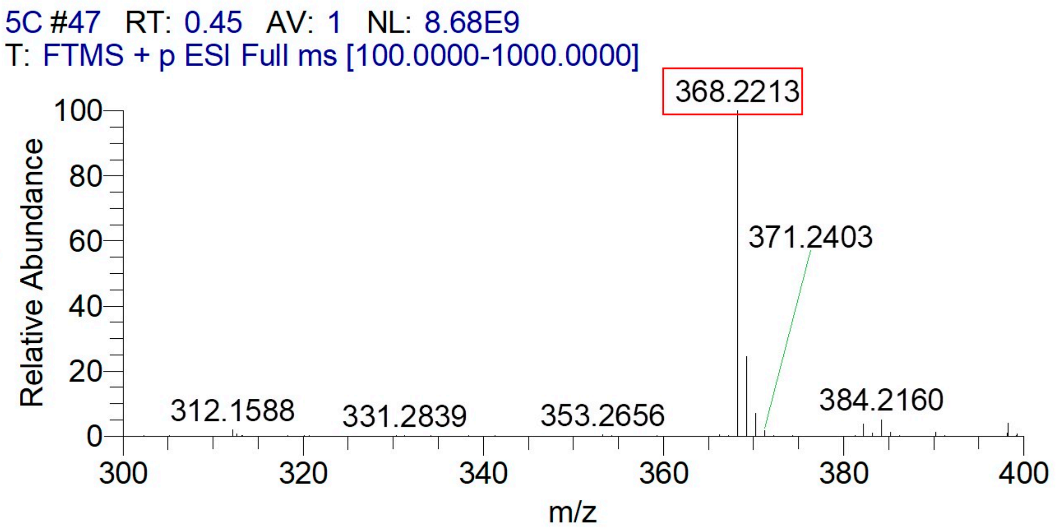

Figure S5. A: Positive MS of paxiphylline D; **B**: Positive MS of reduction reaction product of paxiphylline D (red box) by In.

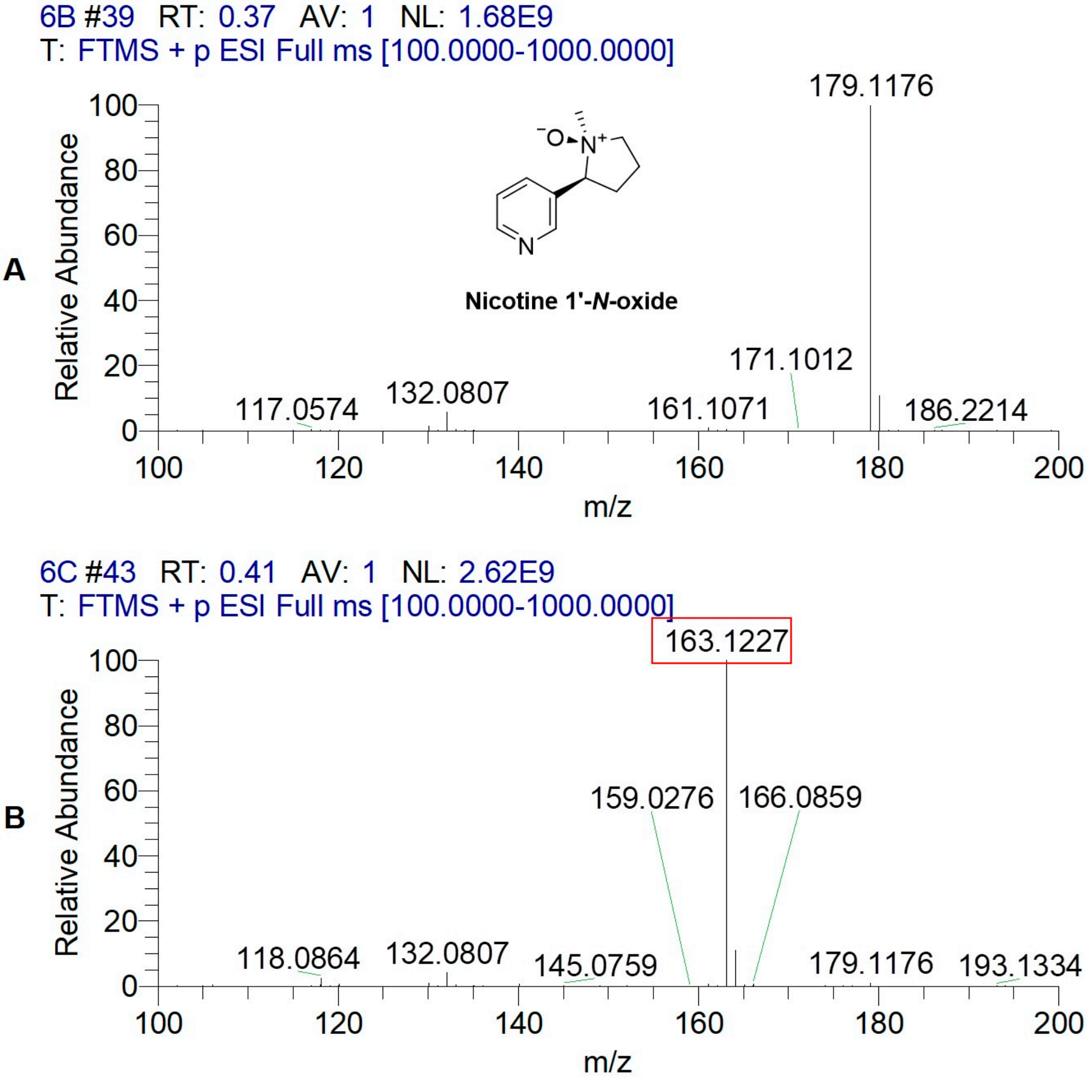

Figure S6. A: Positive MS of nicotine 1'-*N*-oxide; **B**: Positive MS of reduction reaction product of nicotine 1'-*N*-oxide (red box) by In.

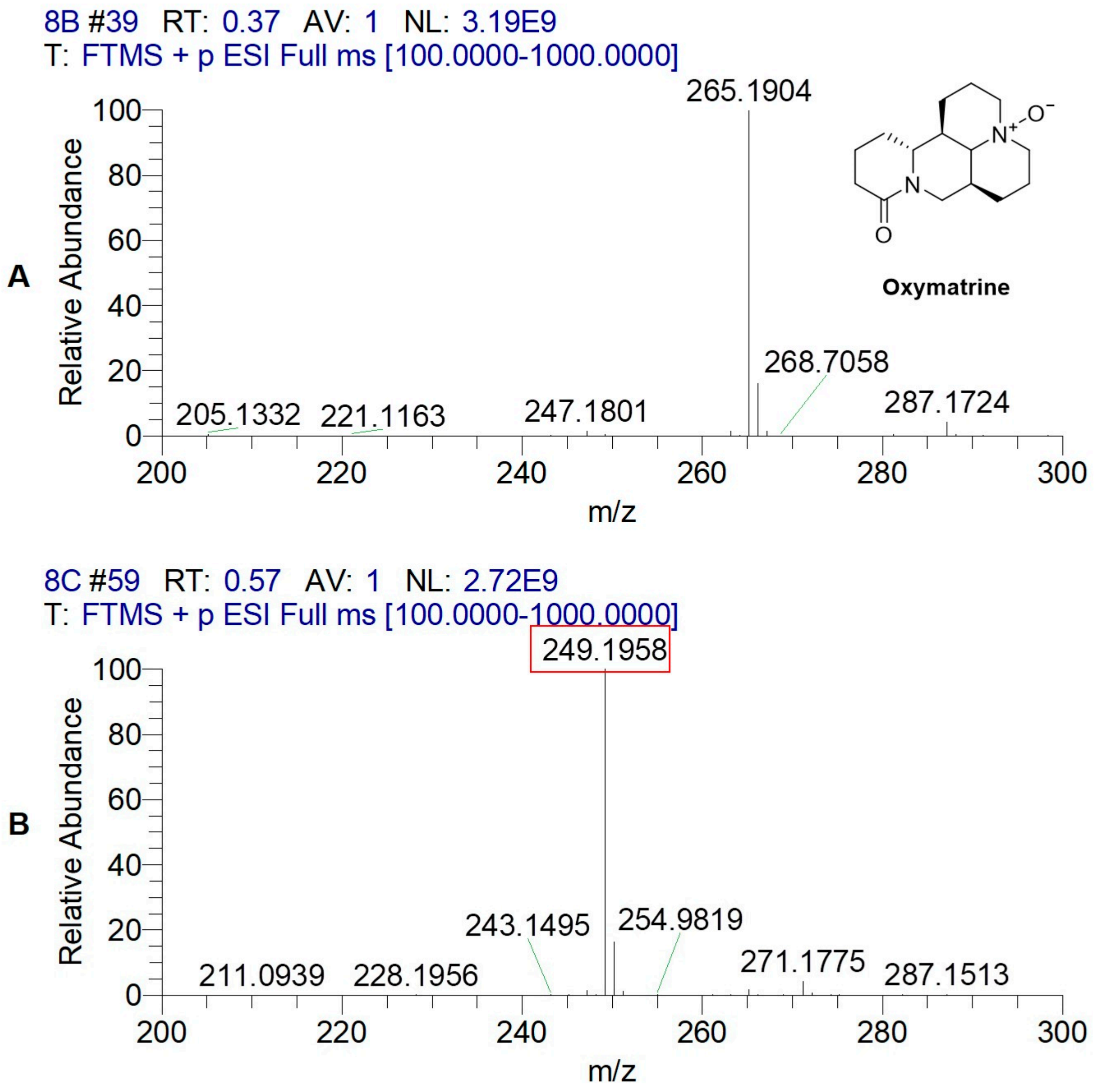

Figure S7. A: Positive MS of oxymatrine; **B**: Positive MS of reduction reaction product of oxymatrine (red box) by In.

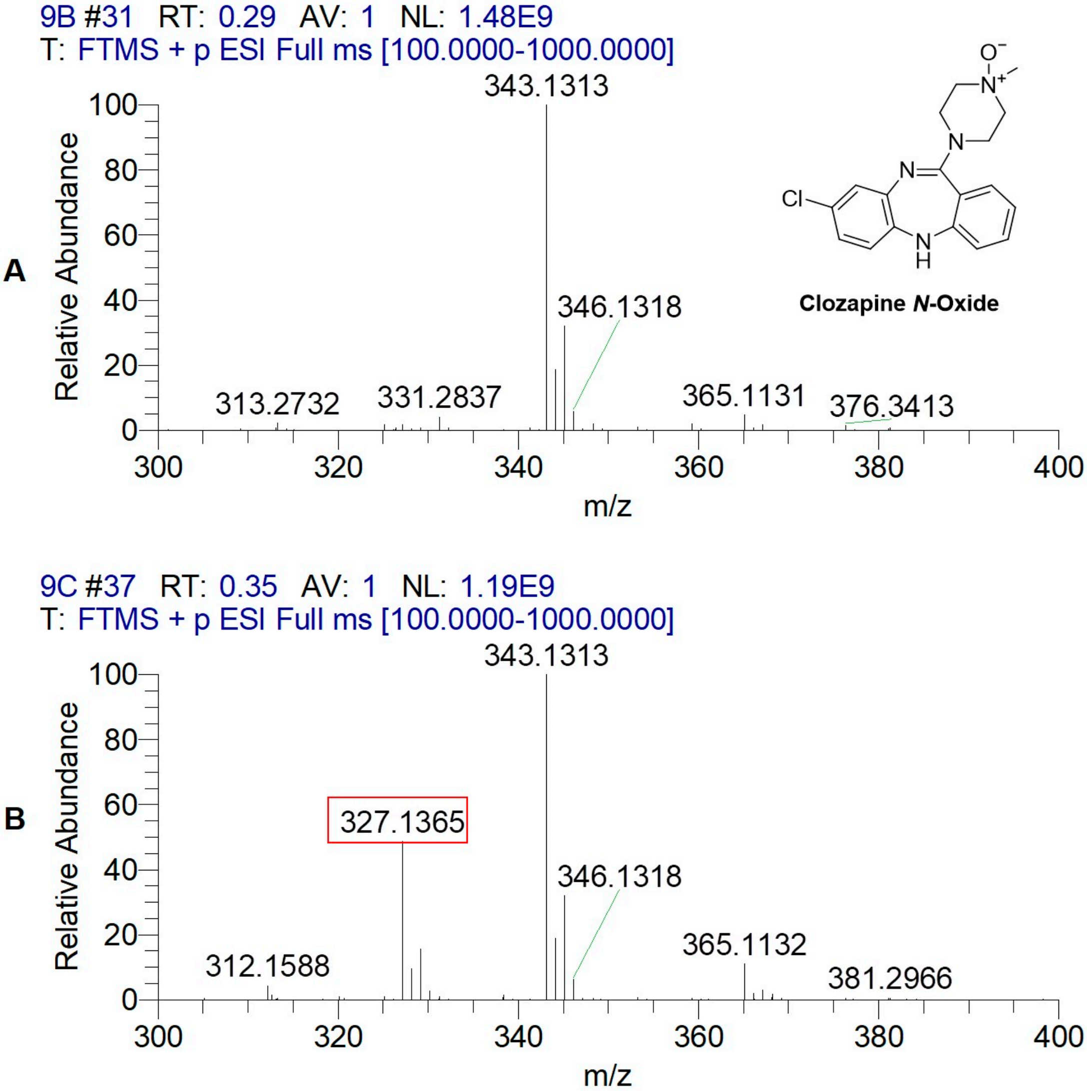

Figure S8. A: Positive MS of clozapine *N*-oxide; **B**: Positive MS of reduction reaction product of clozapine *N*-oxide (red box) by In.

Data S2. NMR, HR-ESI-MS, UV and IR Spectra.

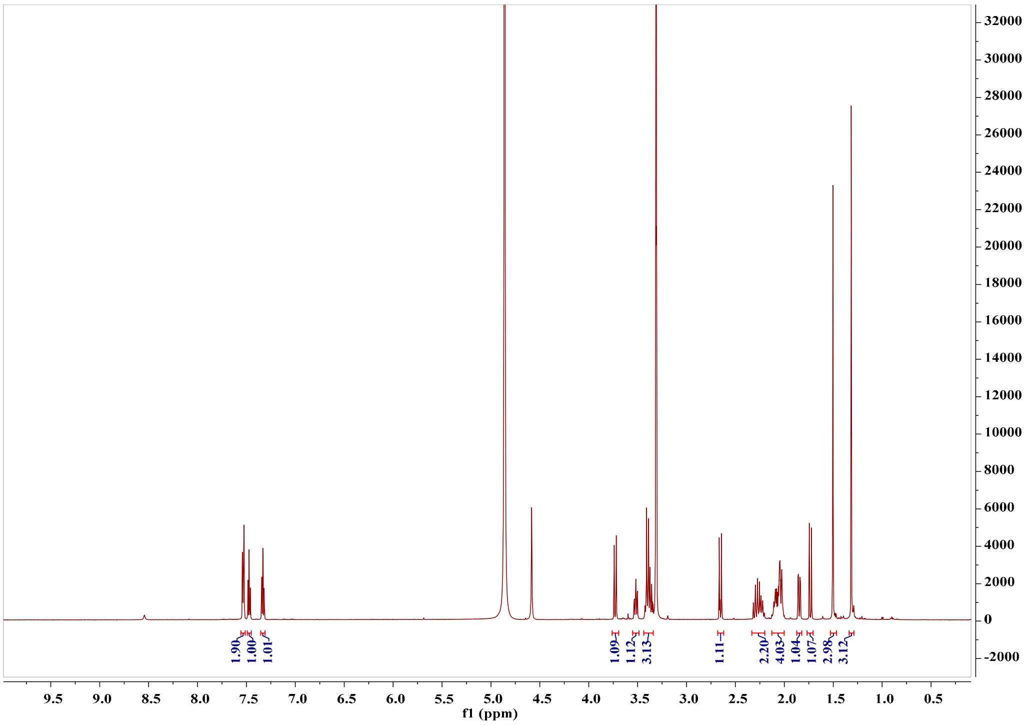

Figure S9. ^{1}H-NMR spectrum of compound **1** (600 MHz in methanol-d_4).

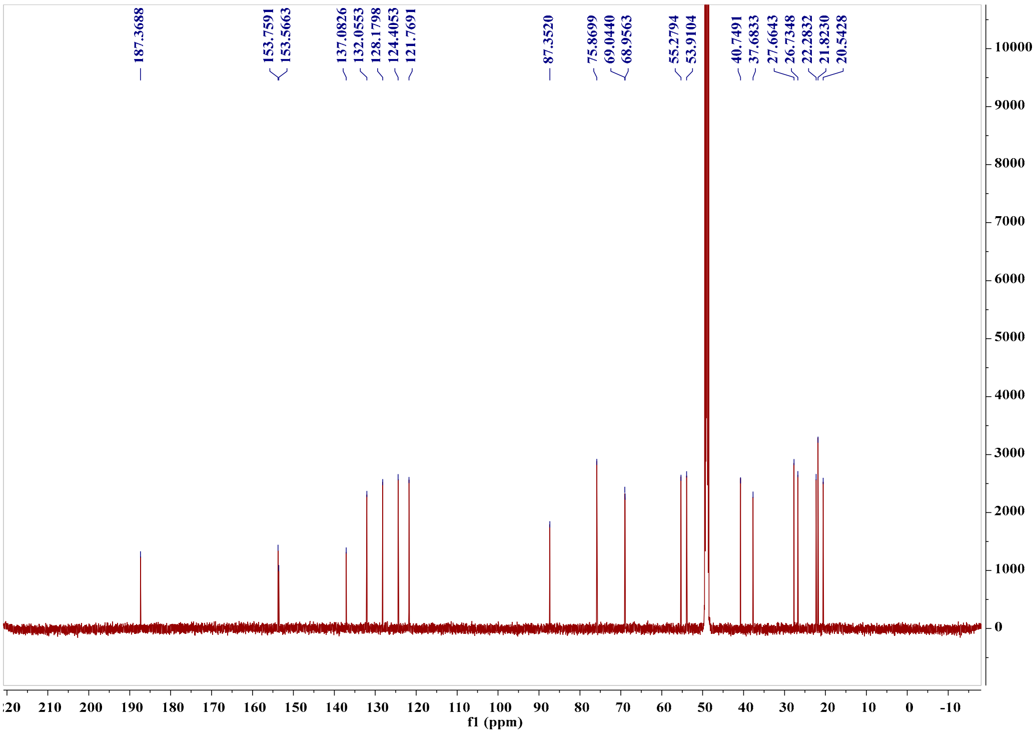

Figure S10. ^{13}C-NMR spectrum of compound **1** (150 MHz in methanol-d_4).

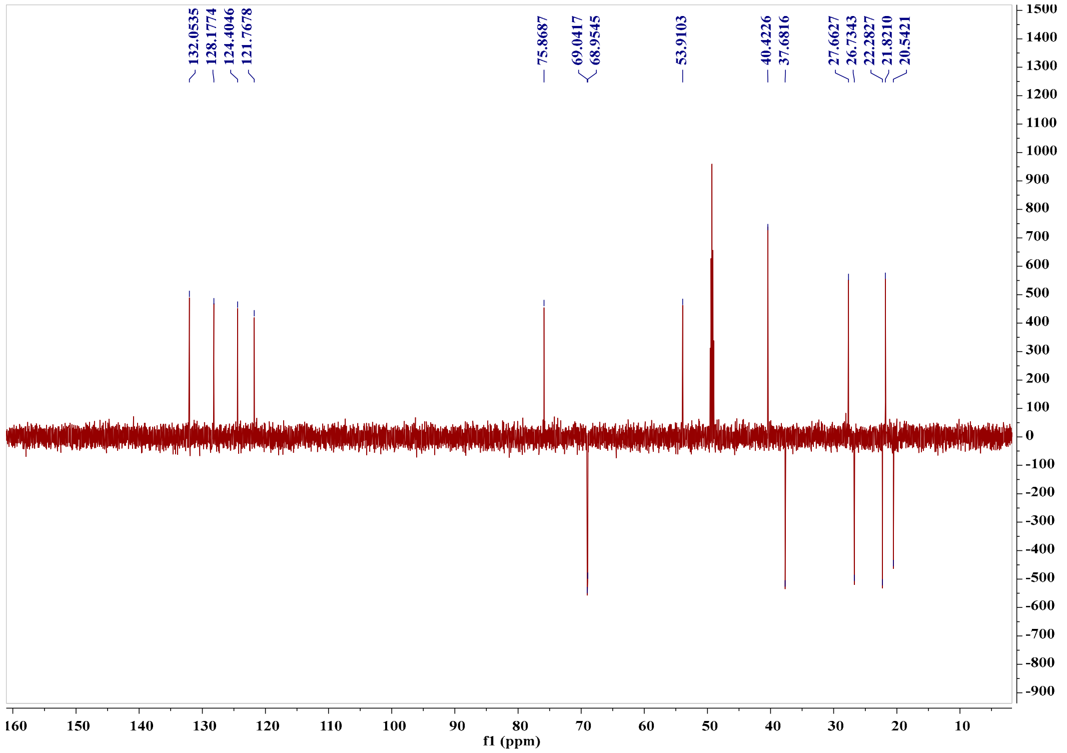

Figure S11. ^{13}C DEPT 135 spectrum of compound **1** (150 MHz in methanol-d_4).

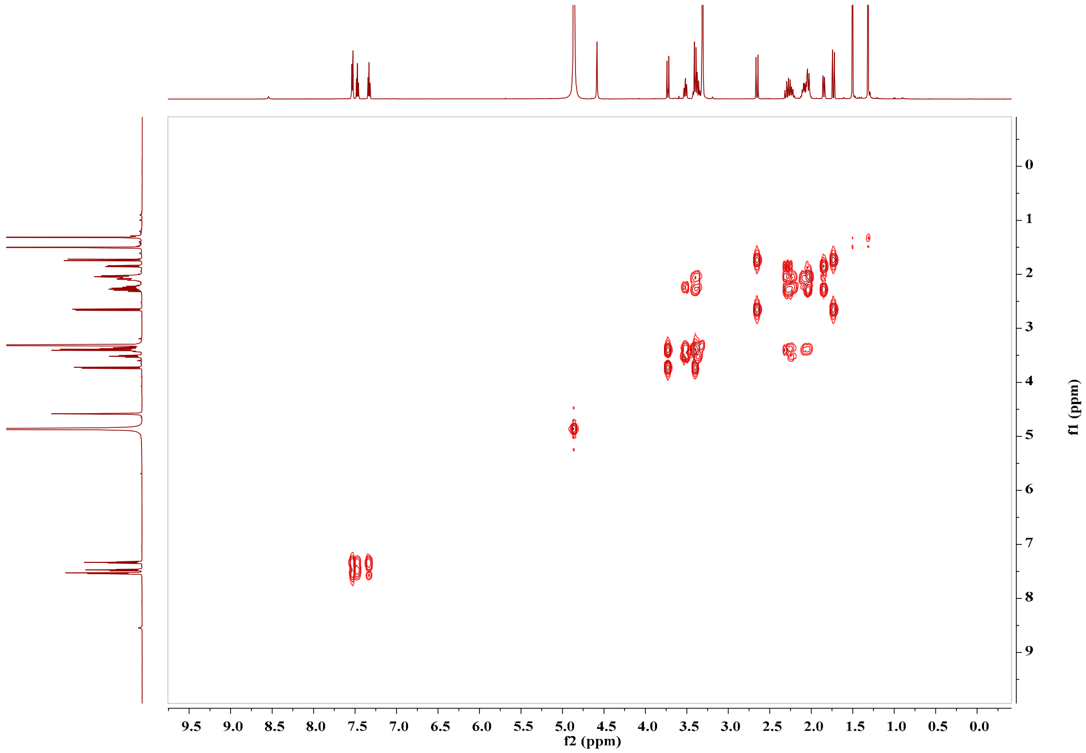

Figure S12. ^{1}H-^{1}H COSY spectrum of compound **1** (in methanol-d_4).

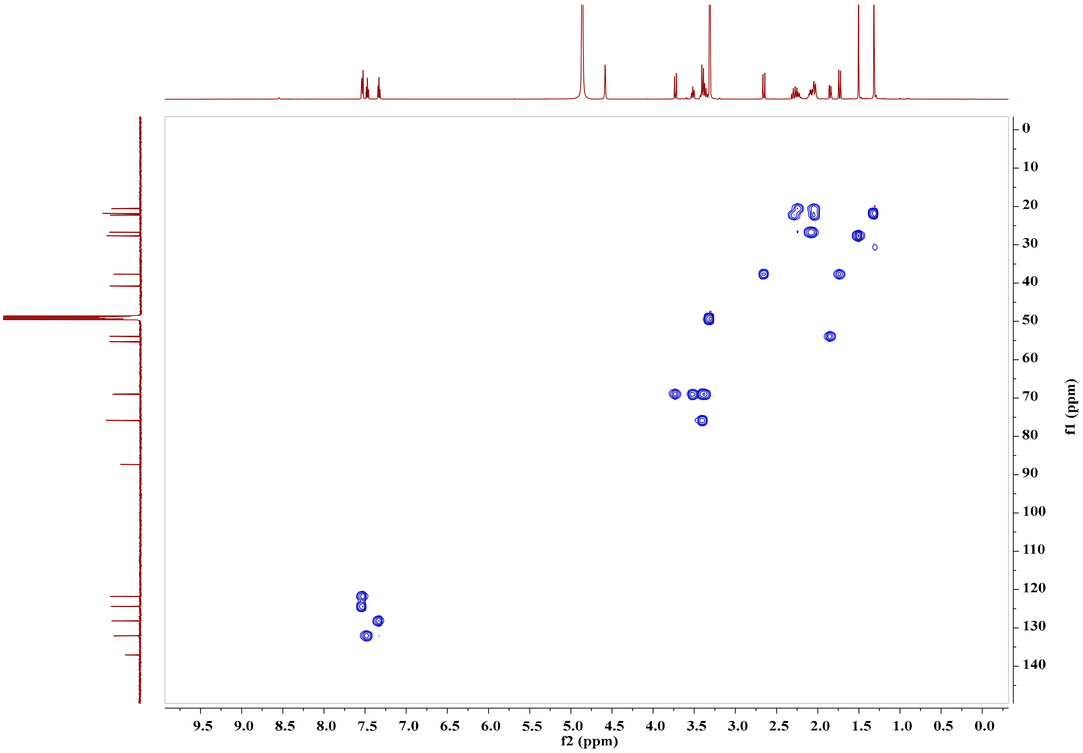

Figure S13. HSQC spectrum of compound **1** (in methanol-d_4).

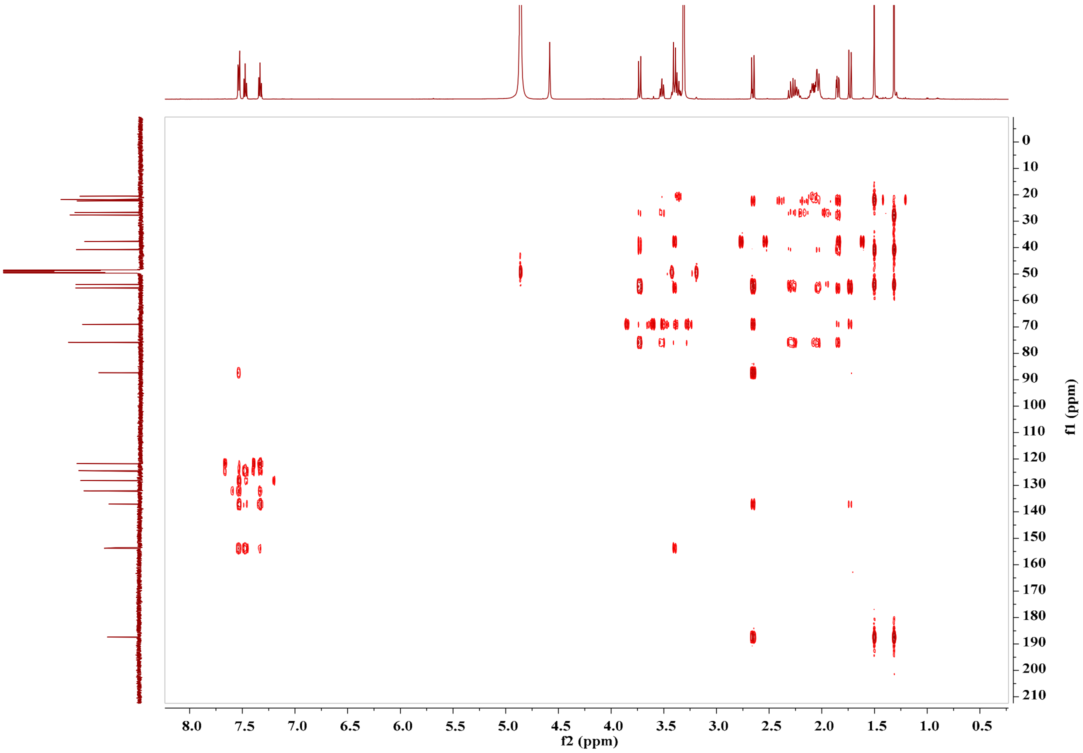

Figure S14. HMBC spectrum of compound **1** (in methanol-d_4).

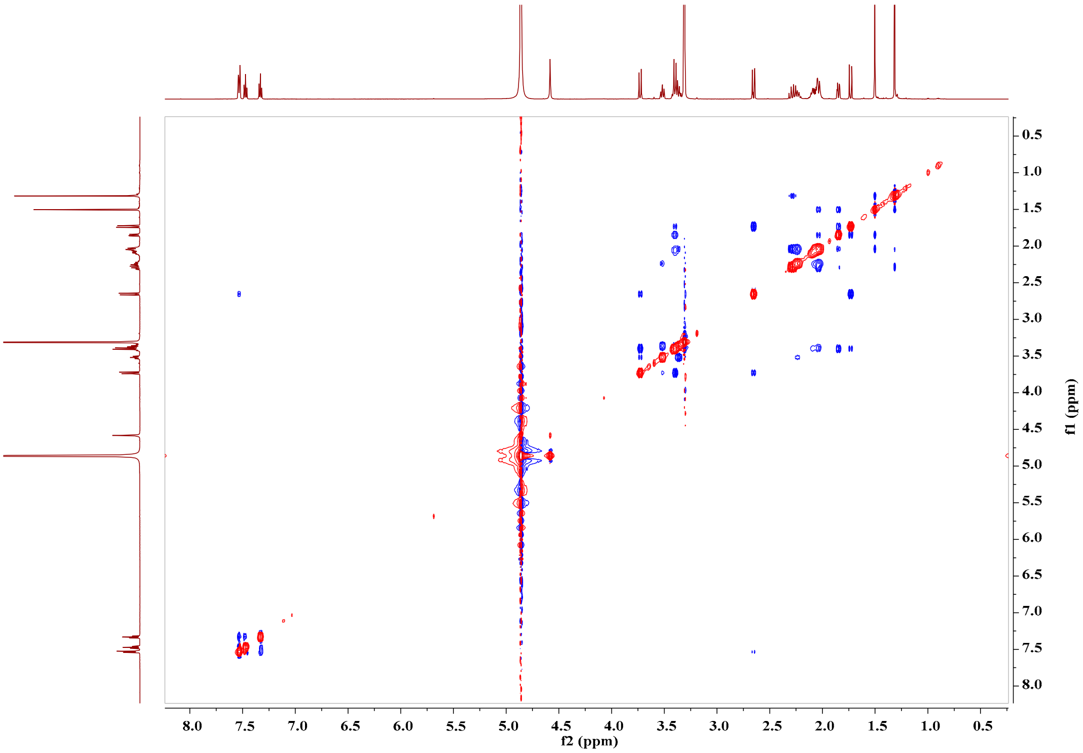

Figure S15. NOESY spectrum of compound **1** (in methanol-d_4).

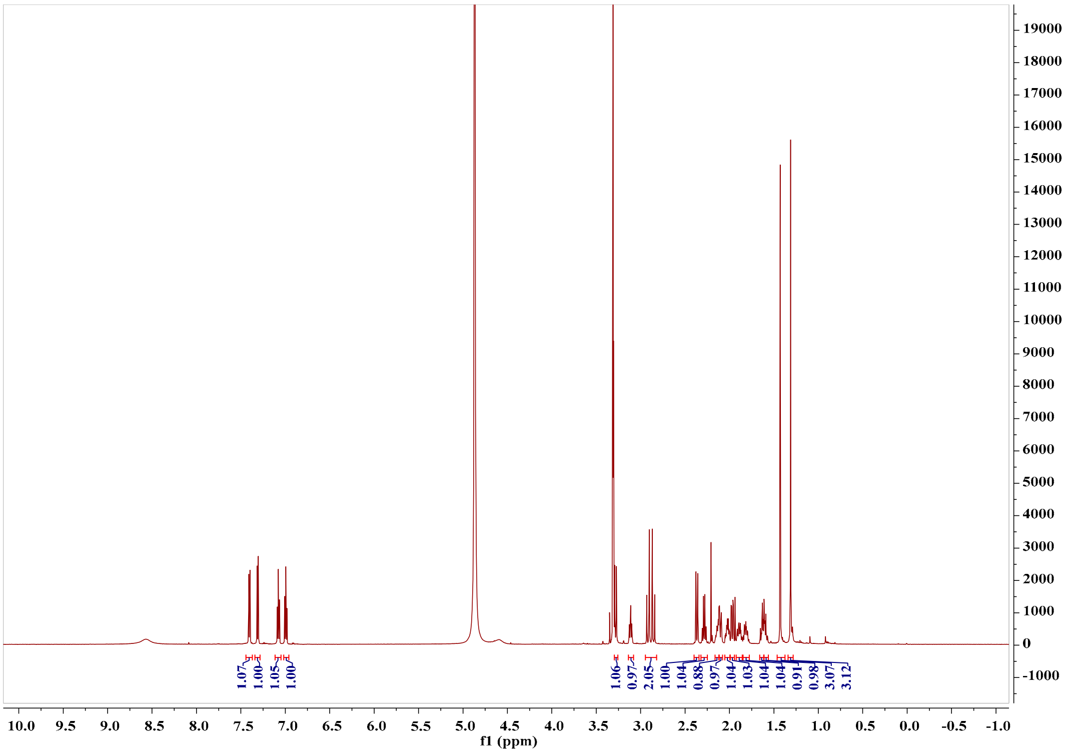

Figure S16. ^{1}H-NMR spectrum of compound **2** (600 MHz in methanol-d_4).

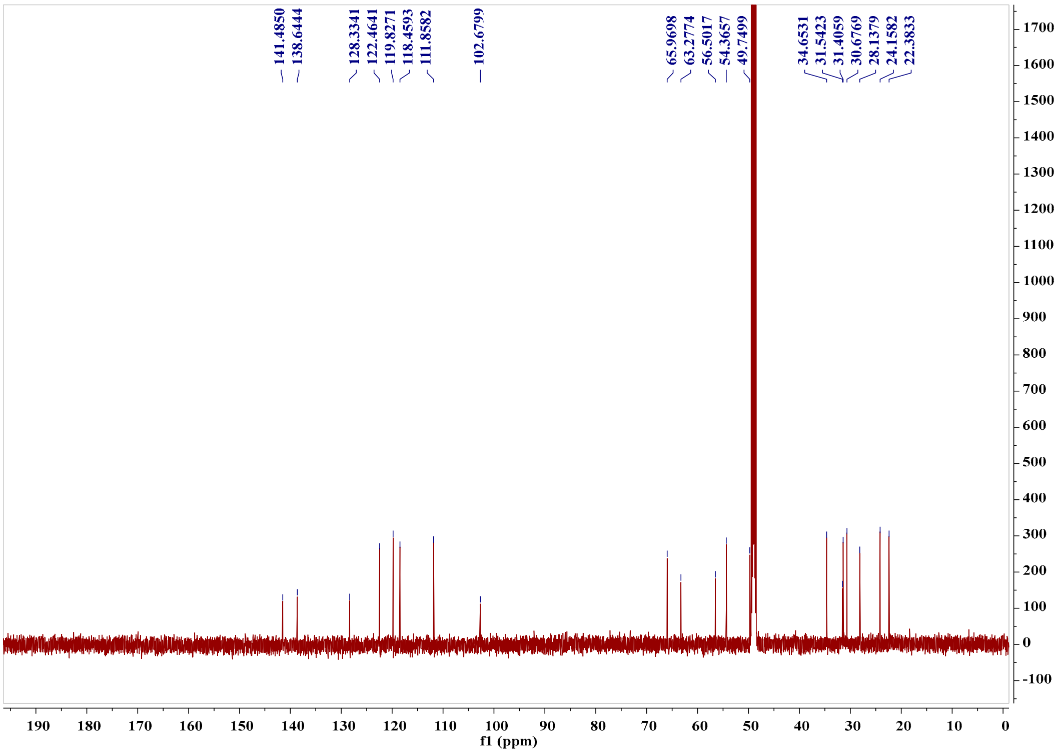

Figure S17. ^{13}C-NMR spectrum of compound **2** (150 MHz in methanol-d_4).

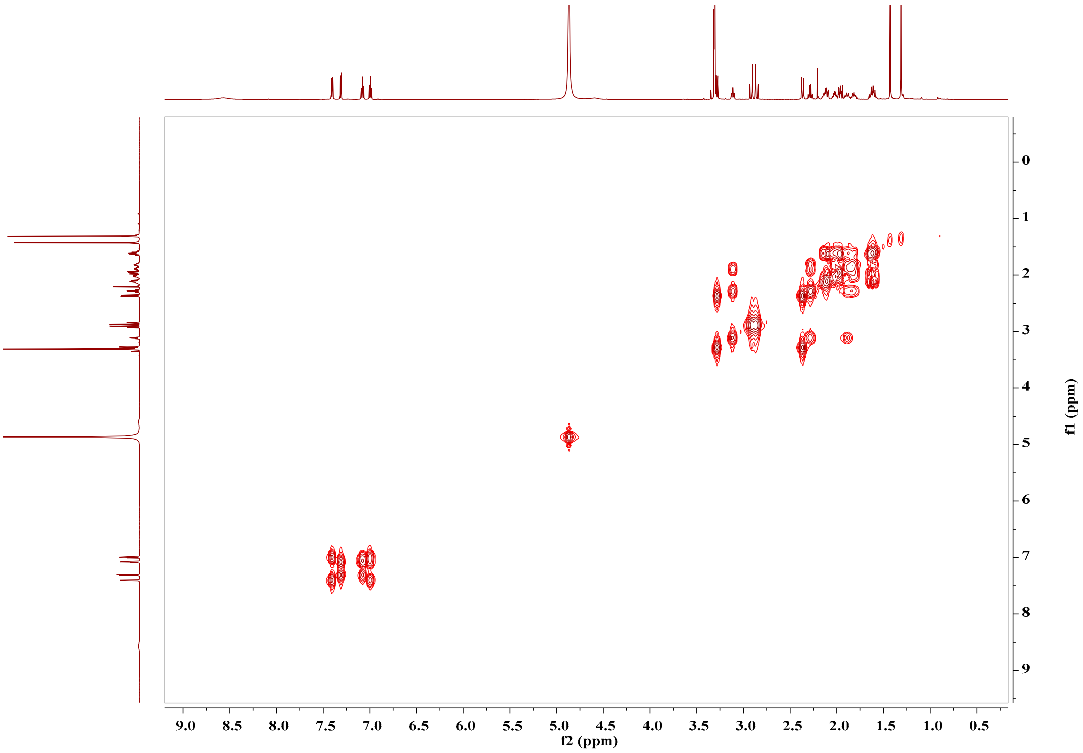

Figure S18. ^{1}H-^{1}H COSY spectrum of compound **2** (in methanol-d_4).

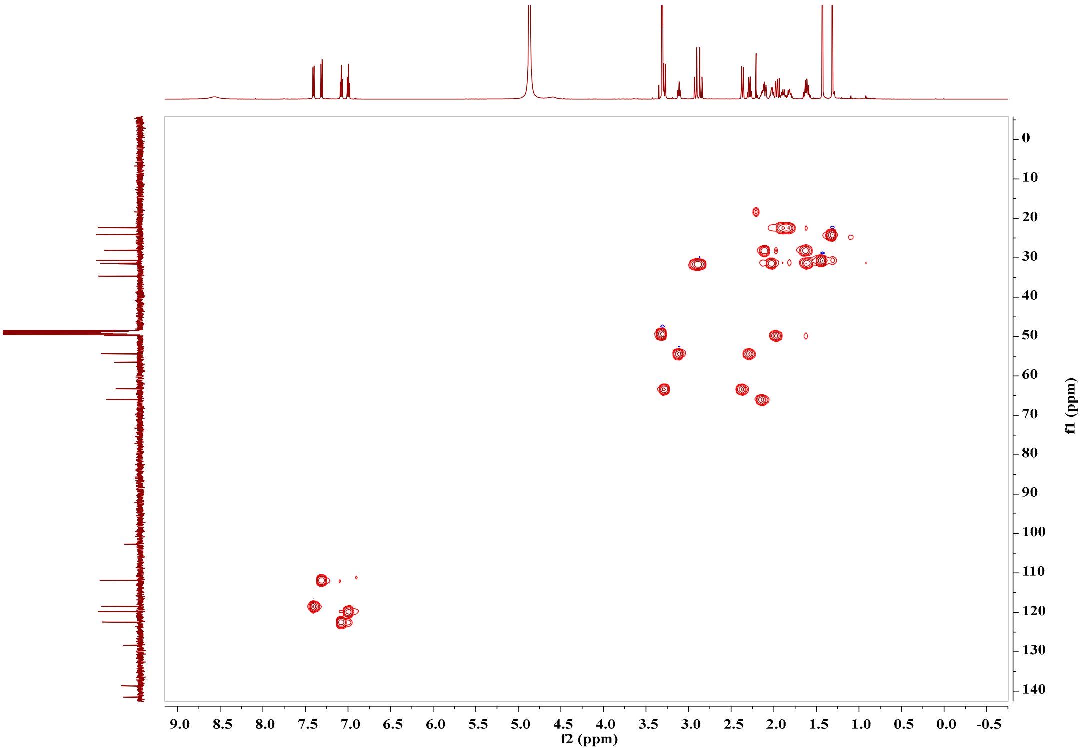

Figure S19. HSQC spectrum of compound **2** (in methanol-d_4).

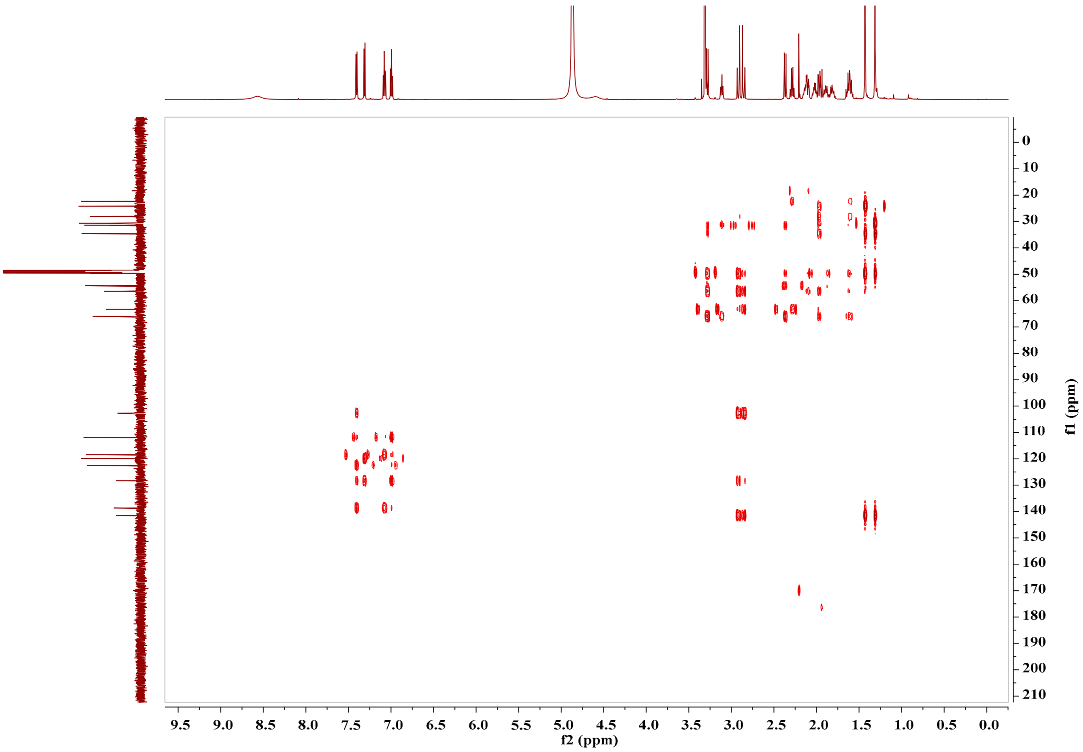

Figure S20. HMBC spectrum of compound **2** (in methanol-d_4).

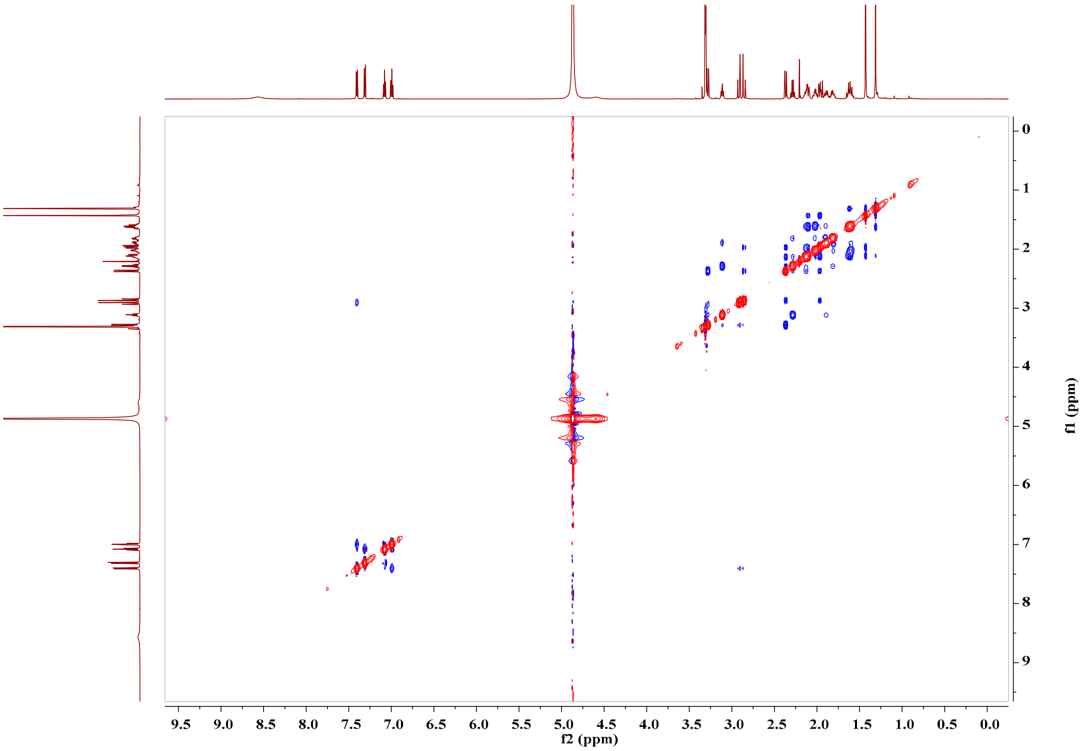

Figure S21. NOESY spectrum of compound **2** (in methanol-d_4).

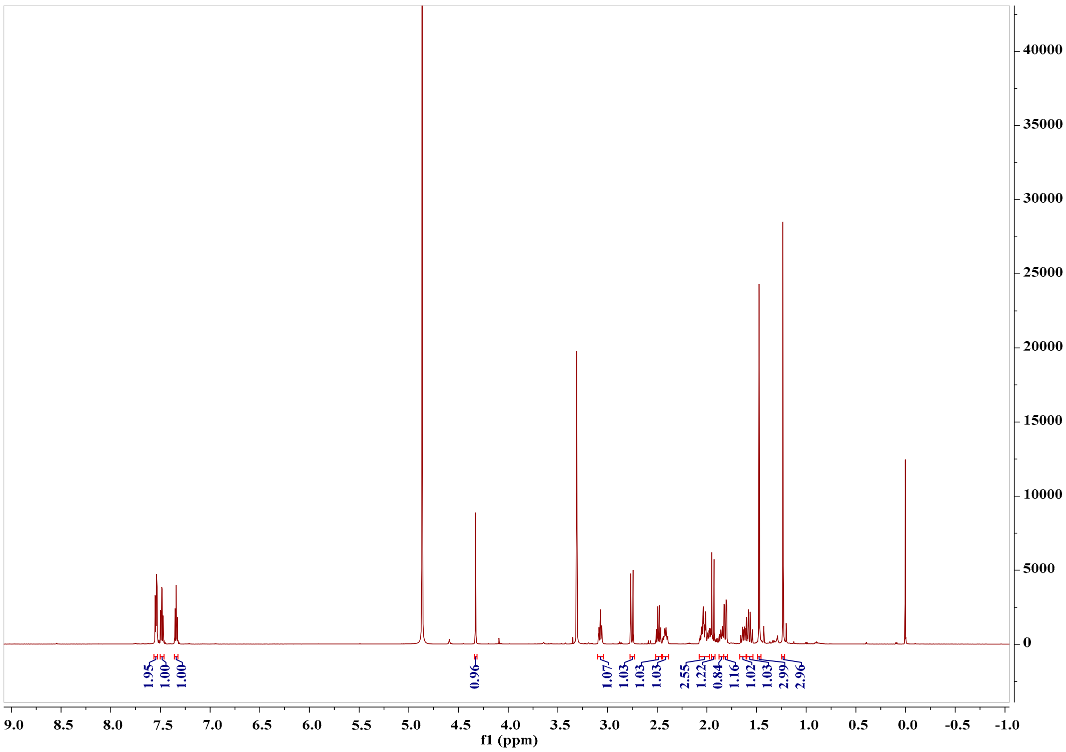

Figure S22. ^{1}H-NMR spectrum of compound **3** (600 MHz in methanol-d_4).

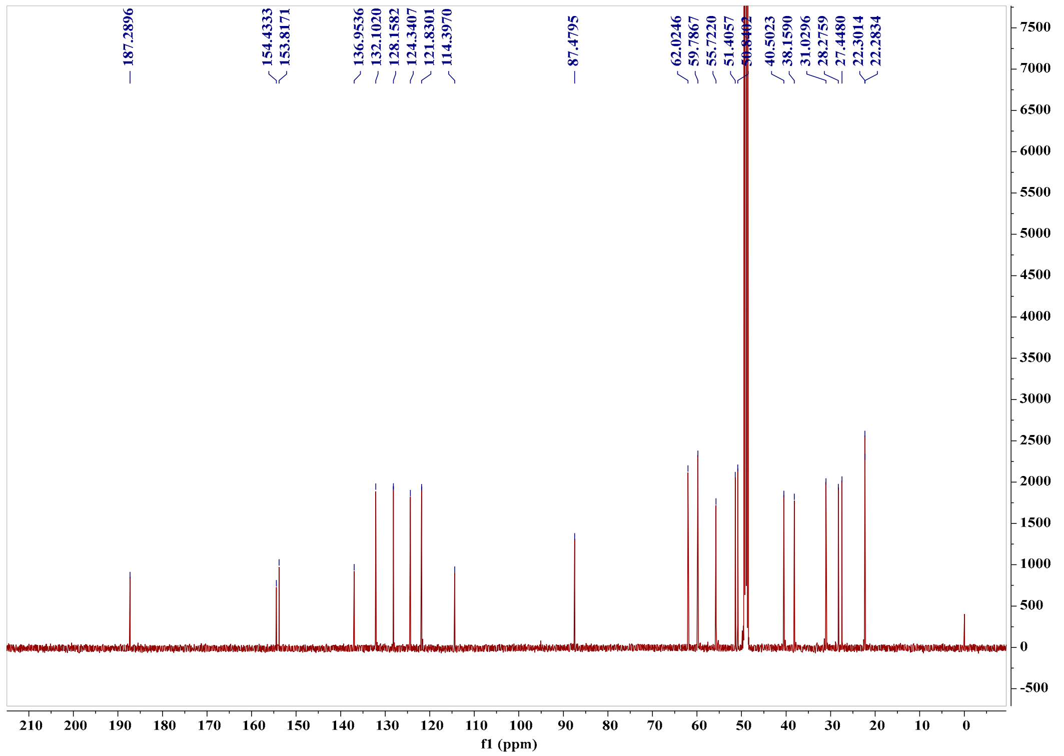

Figure S23. ^{13}C-NMR spectrum of compound **3** (150 MHz in methanol-d_4).

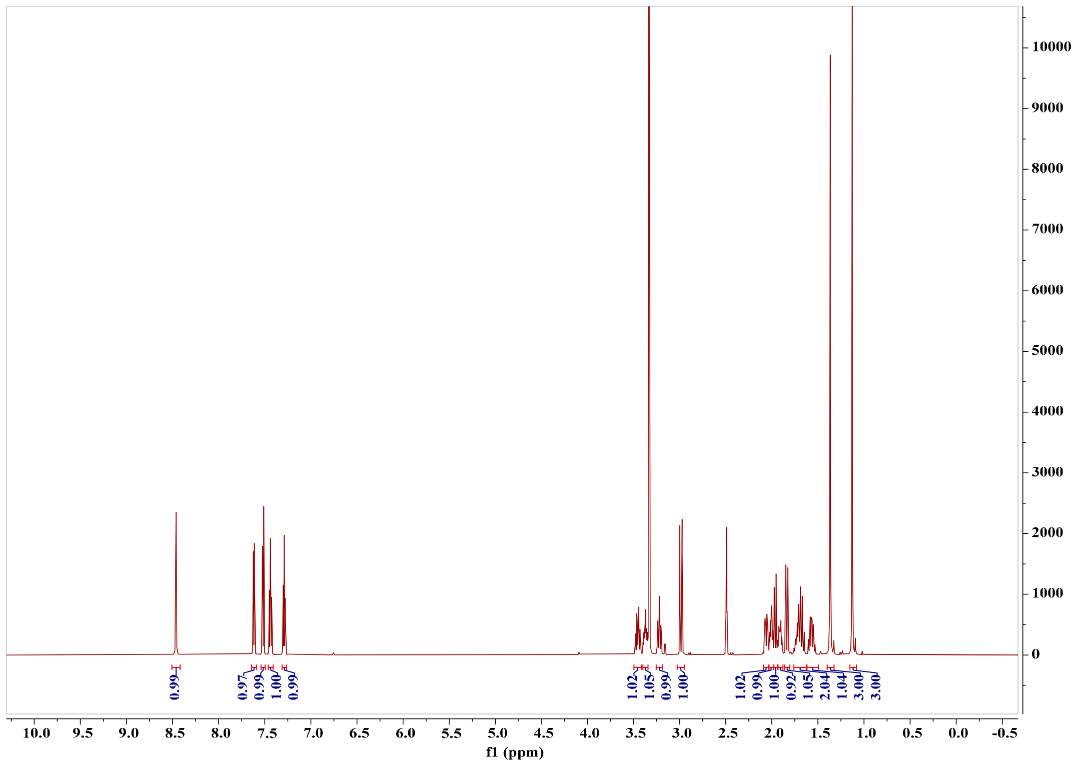

Figure S24. ^{1}H-NMR spectrum of compound **4** (600 MHz in DMSO-d_6).

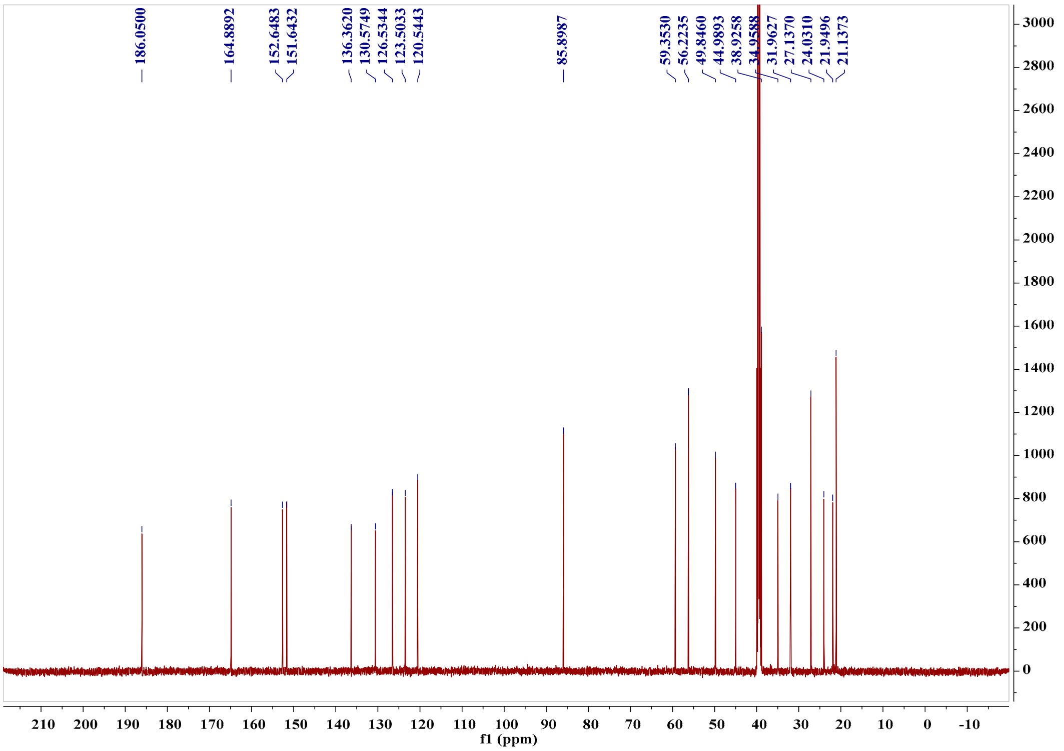

Figure S25. ^{13}C-NMR spectrum of compound **4** (150 MHz in DMSO-d_6).

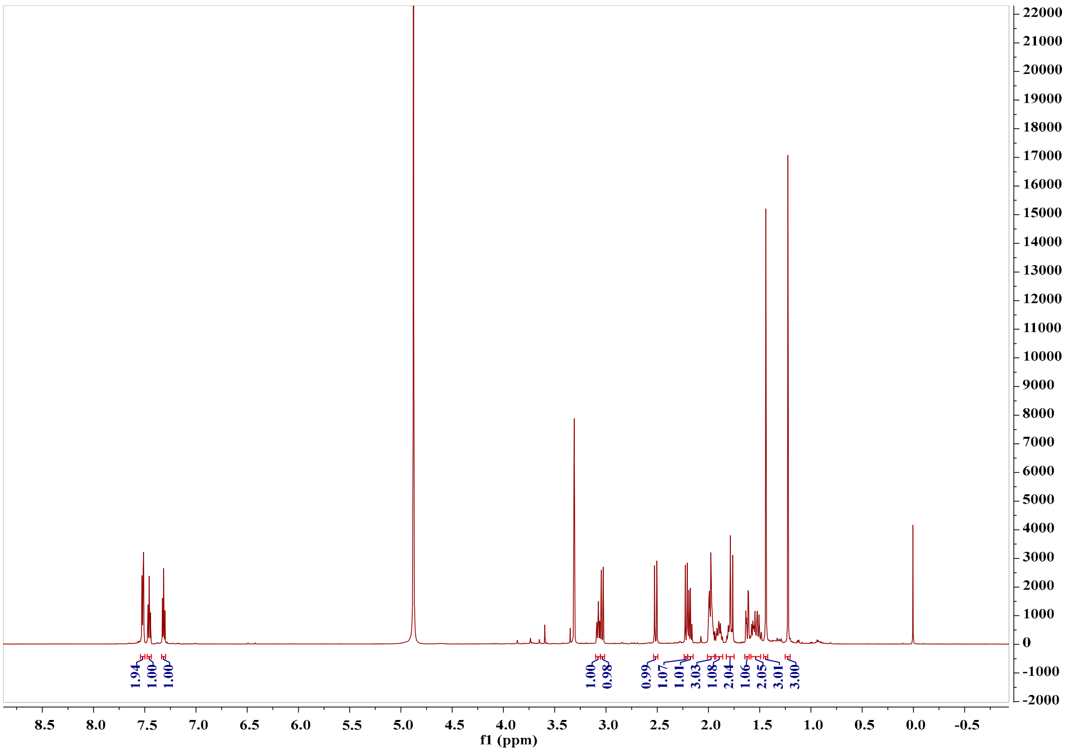

Figure S26. ^{1}H-NMR spectrum of compound **5** (600 MHz in methanol-d_4).

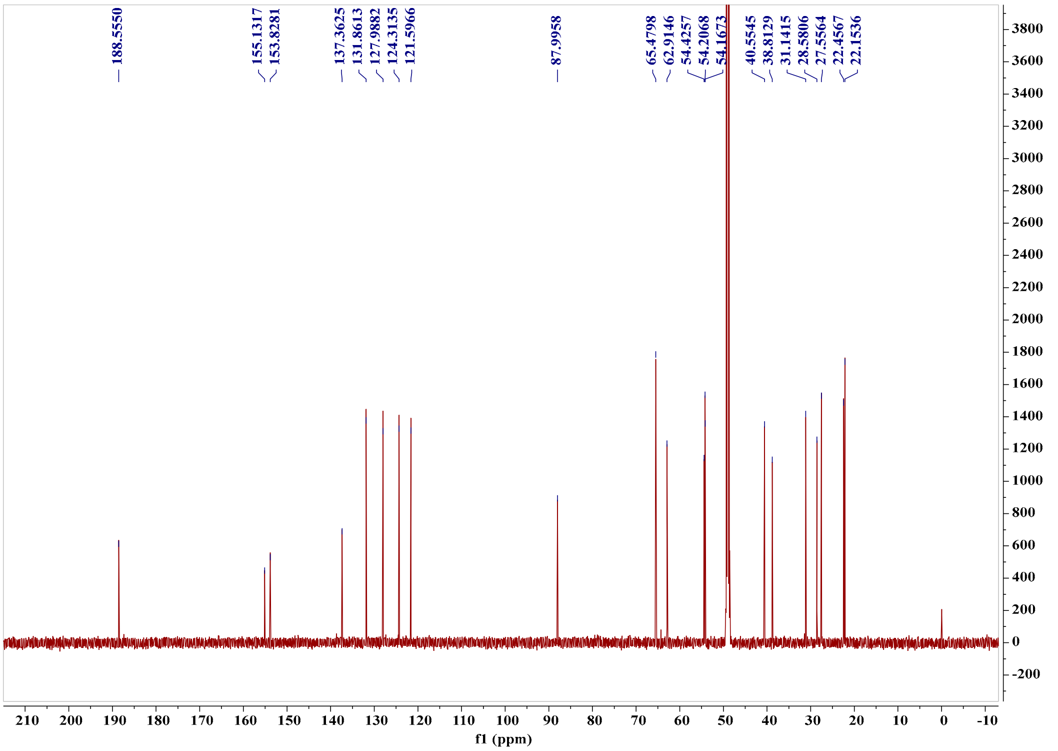

Figure S27. ^{13}C-NMR spectrum of compound **5** (150 MHz in methanol-*d*$_4$).

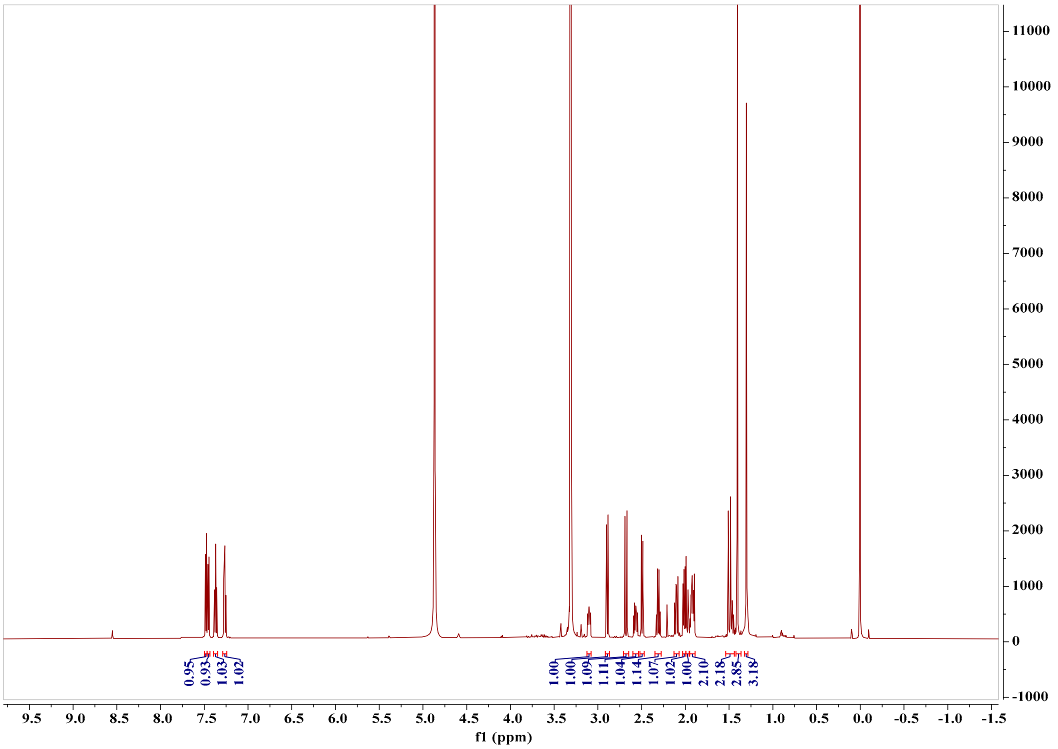

Figure S28. ^{1}H-NMR spectrum of compound **6** (600 MHz in methanol-*d*$_4$).

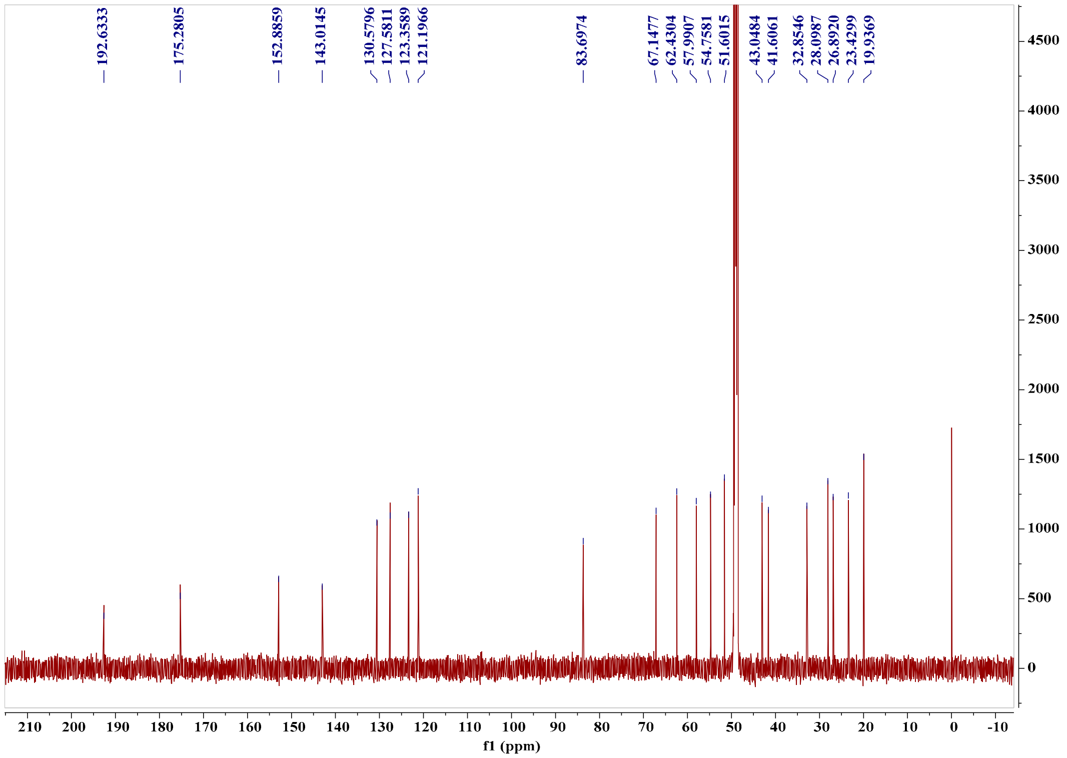

Figure S29. ^{13}C-NMR spectrum of compound **6** (150 MHz in methanol-d_4).

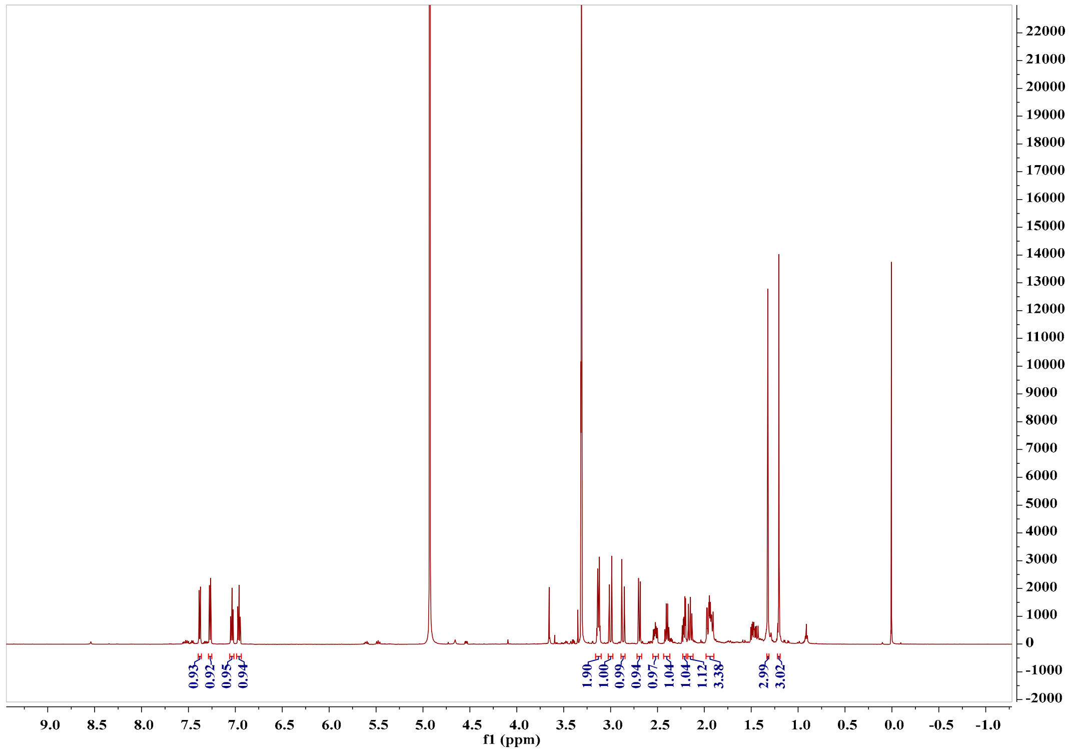

Figure S30. ^{1}H-NMR spectrum of compound **7** (600 MHz in methanol-d_4).

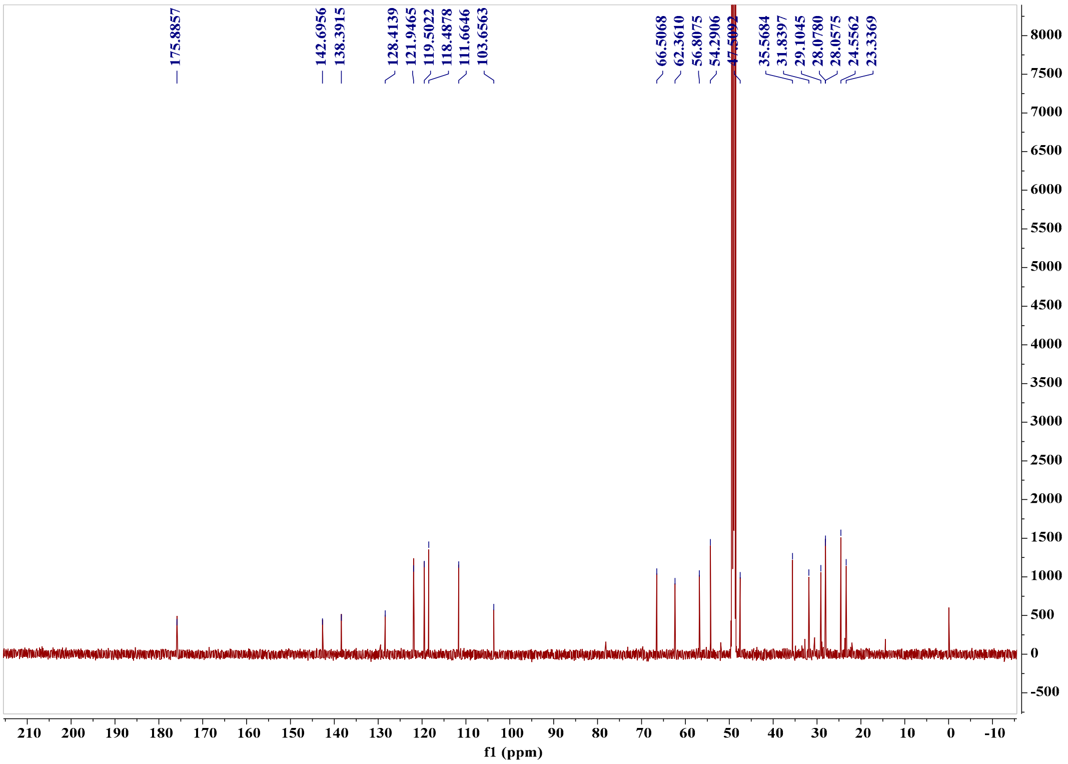

Figure S31. ^{13}C-NMR spectrum of compound **7** (150 MHz in methanol-d_4).

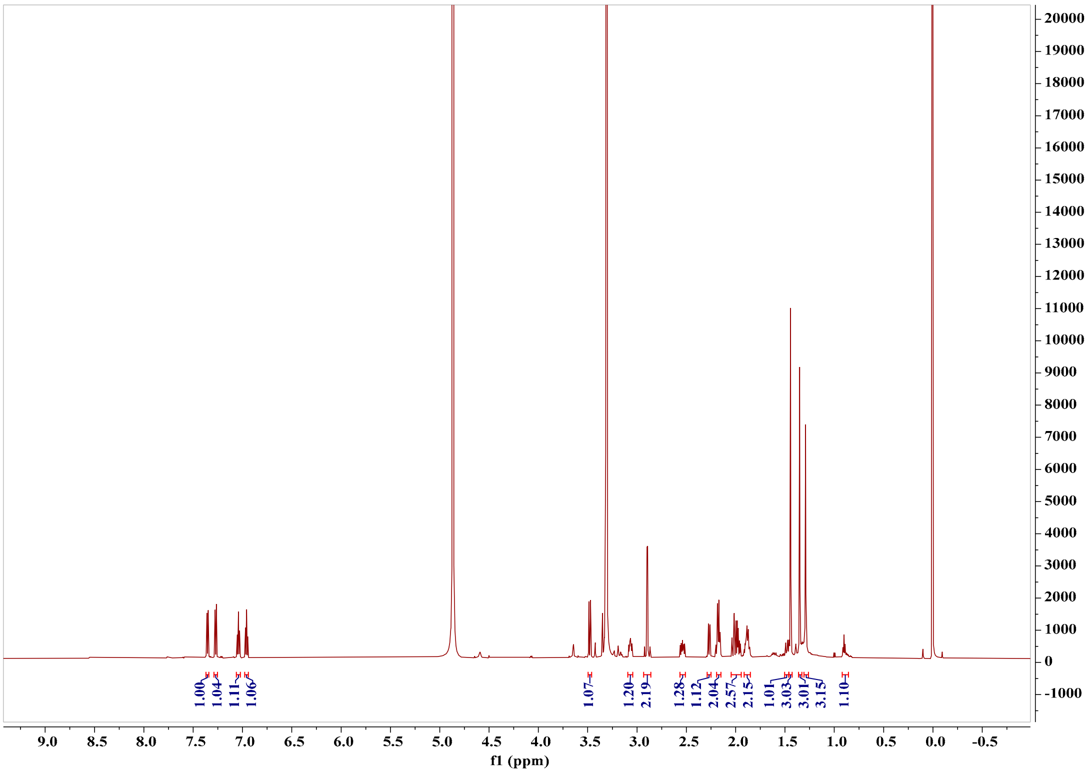

Figure S32. ^{1}H-NMR spectrum of compound **8** (600 MHz in methanol-d_4).

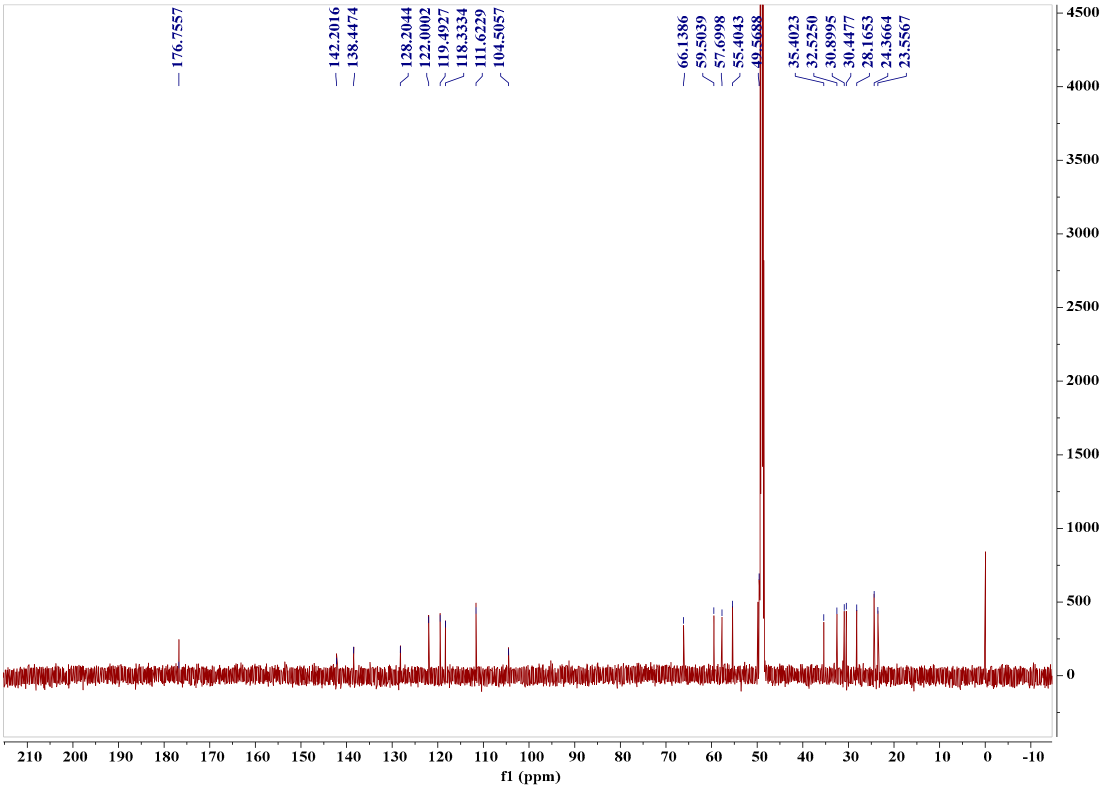

Figure S33. ^{13}C-NMR spectrum of compound **8** (150 MHz in methanol-d_4).

Table S1. ^{1}H-NMR (600 MHz) and ^{13}C-NMR (150 MHz) data for compound **3** and **4**.

No.	Compound **3** (in methanol-d_4)		Compound **4** (in DMSO-d_6)	
	δ_C	δ_H (mult., *J* in Hz)	δ_C	δ_H (mult., *J* in Hz)
2	187.3		186.1	
3	40.5		38.9	
4	50.8	1.81 (1H, dd, *J* = 12.5, 3.6 Hz)	49.9	1.96 (1H, dd, *J* = 12.6, 1.8 Hz)
5α	27.5	1.57 (1H, m)	24.0	1.68 (1H, m)
5β		2.02 (1H, m)		2.06 (1H, ddd, *J* = 13.0, 4.3, 1.9 Hz)
6	59.8	2.42 (1H, m)	59.4	3.37 (1H, m)
7α	31.0	1.63 (1H, m)	32.0	1.57 (1H, qd, *J* = 11.6, 1.8 Hz)
7β		2.06 (1H, m)		2.01 (1H, m)
8α	22.3	1.85 (1H, m)	22.0	1.91 (1H, m)
8β		1.98 (1H, m)		1.72 (1H, m)
9α	51.4	2.49 (1H, q, *J* = 8.8 Hz)	45.0	3.45 (1H, dt, *J* = 12.4, 8.8 Hz)
9β		3.07 (1H, td, *J* = 8.7, 2.8 Hz)		3.22 (1H, m)
11	62.0	4.33 (1H, s)	164.9	
12	55.7		56.2	
13α	38.2	1.94 (1H, d, *J* = 13.7 Hz)	35.0	2.98 (1H, d, *J* = 13.7 Hz)
13β		2.75 (1H, d, *J* = 13.7 Hz)		1.84 (1H, dd, *J* = 13.7, 1.4 Hz)
14	87.5		85.9	
15	137.0		136.4	
16	124.3	7.54 (1H, br d, *J* = 7.5 Hz)	123.5	7.62 (1H, br d, *J* = 7.1 Hz)
17	128.2	7.34 (1H, td, *J* = 7.4, 1.0 Hz)	126.5	7.29 (1H, td, *J* = 7.4, 1.0 Hz)
18	132.1	7.49 (1H, td, *J* = 7.6, 1.1 Hz)	130.6	7.44 (1H, td, *J* = 7.6, 1.3 Hz)
19	121.8	7.55 (1H, br d, *J* = 7.8 Hz)	120.5	7.52 (1H, br d, *J* = 7.6 Hz)
20	154.4		152.7	
21	22.3	1.23 (3H, s)	27.1	1.36 (3H, s)
22	28.3	1.47 (3H, s)	21.1	1.13 (3H, s)
23	114.4		151.6	
25	153.8			

Table S2. ^{1}H-NMR (600 MHz) and ^{13}C-NMR (150 MHz) data for compound **5** (in methanol-d_4).

No.	δ_C	δ_H (mult., *J* in Hz)
2	188.6	
3	40.6	
4	54.1	1.62 (1H, dd, *J* = 12.4, 3.1 Hz)
5α	28.6	1.51 (1H, td, *J* = 13.0, 10.4 Hz)
5β		2.00-1.94 (1H, m)
6	65.5	2.00-1.94 (1H, m)
7α	31.1	1.59-1.54 (1H, m)
7β		2.00-1.94 (1H, m)
8α	22.5	1.93-1.86 (1H, m)
8β		1.83-1.76 (1H, m)
9α	54.2	3.07 (1H, td, *J* = 8.7, 2.2 Hz)
9β		2.18 (1H, q, *J* = 8.9 Hz)
11α	62.9	3.04 (1H, d, *J* = 11.2 Hz)
11β		2.22 (1H, d, *J* = 11.2 Hz)
12	54.4	
13α	38.8	2.52 (1H, d, *J* = 13.9 Hz)
13β		1.77 (1H, d, *J* = 14.1 Hz)
14	88.0	
15	137.4	
16	124.3	7.521 (1H, br d, *J* = 7.3 Hz)
17	128.0	7.31 (1H, td, *J* = 7.6, 1.0 Hz)
18	131.9	7.46 (1H, td, *J* = 7.6, 0.9 Hz)
19	121.6	7.517 (1H, br d, *J* = 7.4 Hz)
20	153.8	
21	27.6	1.44 (3H, s)
22	22.2	1.22 (3H, s)
23	155.1	

Table S3. ^{1}H-NMR (600 MHz) and ^{13}C-NMR (150 MHz) data for compound **6** (in methanol-d_4)

No.	δ_C	δ_H (mult., J in Hz)
2	192.6	
3	41.6	
4	51.6	2.00 (1H, dd, J = 9.3, 5.0 Hz)
5α	32.9	2.10 (1H, dd, J = 12.7, 9.2 Hz)
5β		1.98 (1H, dd, J = 12.7, 5.1 Hz)
6	67.2	
7α	28.1	2.57 (1H, dt, J = 14.3, 6.5 Hz)
7β		1.47 (1H, td, J = 12.7, 9.0 Hz)
8	23.4	1.92 (2H, m)
9α	54.7	3.10 (1H, dt, J = 11.4, 5.3 Hz)
9β		2.31 (1H, q, J = 8.8 Hz)
11α	62.4	2.89 (1H, d, J = 10.3 Hz)
11β		2.49 (1H, d, J = 10.3 Hz)
12	58.0	
13α	43.1	2.68 (1H, d, J = 14.9 Hz)
13β		1.49 (1H, d, J = 14.9 Hz)
14	83.7	
15	143.0	
16	123.3	7.45 (1H, br d, J = 7.3 Hz)
17	127.6	7.26 (1H, td, J = 7.4, 1.0 Hz)
18	130.6	7.37 (1H, td, J = 7.6, 1.3 Hz)
19	121.2	7.48 (1H, br d, J = 7.7 Hz)
20	152.9	
21	26.9	1.30 (3H, s)
22	19.9	1.41 (3H, s)
23	175.3	

Table S4. ^{1}H-NMR (600 MHz) and ^{13}C-NMR (150 MHz) data for compound **7** and **8**.

No.	Compound **7** (in methanol-d_4)		Compound **8** (in methanol-d_4)	
	δ_C	δ_H (mult., *J* in Hz)	δ_C	δ_H (mult., *J* in Hz)
2	142.7		142.2	
3	31.8		35.4	
4	47.5	2.21 (1H, dd, *J* = 10.1, 3.8 Hz)	48.8	2.17 (1H, dd, *J* = 10.9, 3.2 Hz)
5α	31.8	2.15 (1H, dd, *J* = 13.6, 10.1 Hz)	32.5	2.06 (1H, dd, *J* = 13.1, 10.9 Hz)
5β		1.96 (1H, dd, *J* = 13.6, 3.8 Hz)		1.97 (1H, dd, *J* = 13.2, 5.1 Hz)
6	66.5		66.1	
7α	28.1	2.52 (1H, ddd, *J* = 13.9, 8.2, 5.1 Hz)	28.2	2.54 (1H, m)
7β		1.48 (1H, m)		1.47 (1H, m)
8	23.3	1.94-1.90 (2H, m)	23.6	1.91-1.96 (2H, m)
9α	54.3	3.13 (1H, m)	55.4	3.07 (1H, m)
9β		2.40 (1H, q, *J* = 8.7 Hz)		2.18 (1H, br q, *J* = 8.8 Hz)
11α	62.4	3.12 (1H, d, *J* = 10.6 Hz)	59.5	3.48 (1H, d, *J* = 10.3 Hz)
11β		2.69 (1H, d, *J* = 10.4 Hz)		2.27 (1H, dd, *J* = 10.3, 1.8 Hz)
12	56.8		57.7	
13α	29.1	3.00 (1H, d, *J* = 16.9 Hz)	30.5	2.91 (1H, d, *J* = 15.4 Hz)
13β		2.87 (1H, d, *J* = 16.9 Hz)		2.88 (1H, d, *J* = 15.4 Hz)
14	103.7		104.5	
15	128.4		128.2	
16	118.5	7.38 (1H, br d, *J* = 7.7 Hz)	118.3	7.35 (1H, br d, *J* = 7.7 Hz)
17	119.5	6.96 (1H, td, *J* = 7.0, 1.1 Hz)	119.5	6.96 (1H, td, *J* = 7.0, 1.0 Hz)
18	122.0	7.04 (1H, td, *J* = 7.1, 1.2 Hz)	122.0	7.04 (1H, td, *J* = 7.1, 1.1 Hz)
19	111.7	7.27 (1H, br d, *J* = 8.0 Hz)	111.6	7.27 (1H, br d, *J* = 8.0 Hz)
20	138.4		138.5	
21	28.1	1.32 (3H, s)	24.4	1.44 (3H, s)
22	24.6	1.21 (3H, s)	30.9	1.35 (3H, s)
23	175.9		176.8	

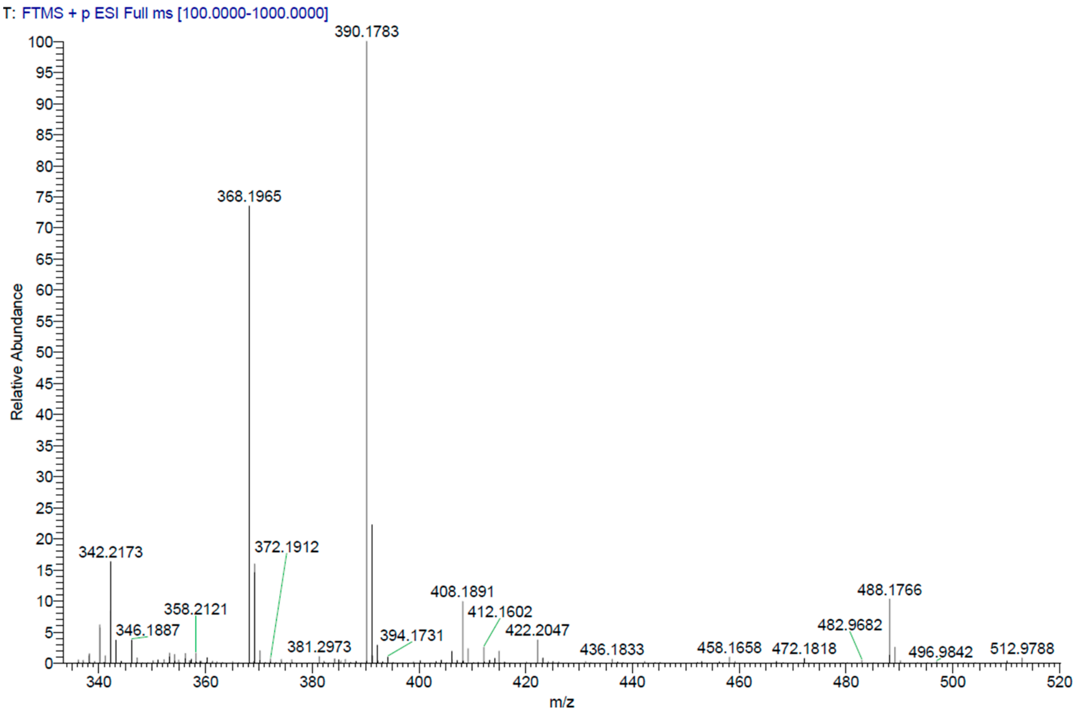

Figure S34. (+) HR-ESI-MS spectrum of compound **1**.

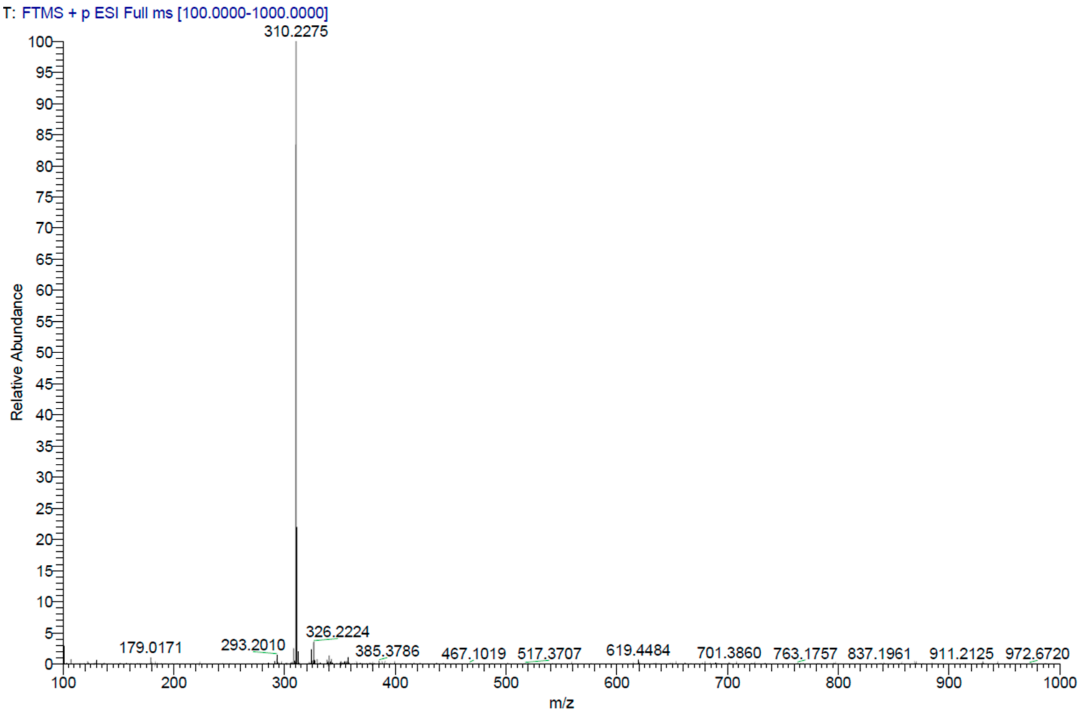

Figure S35. (+) HR-ESI-MS spectrum of compound **2**.

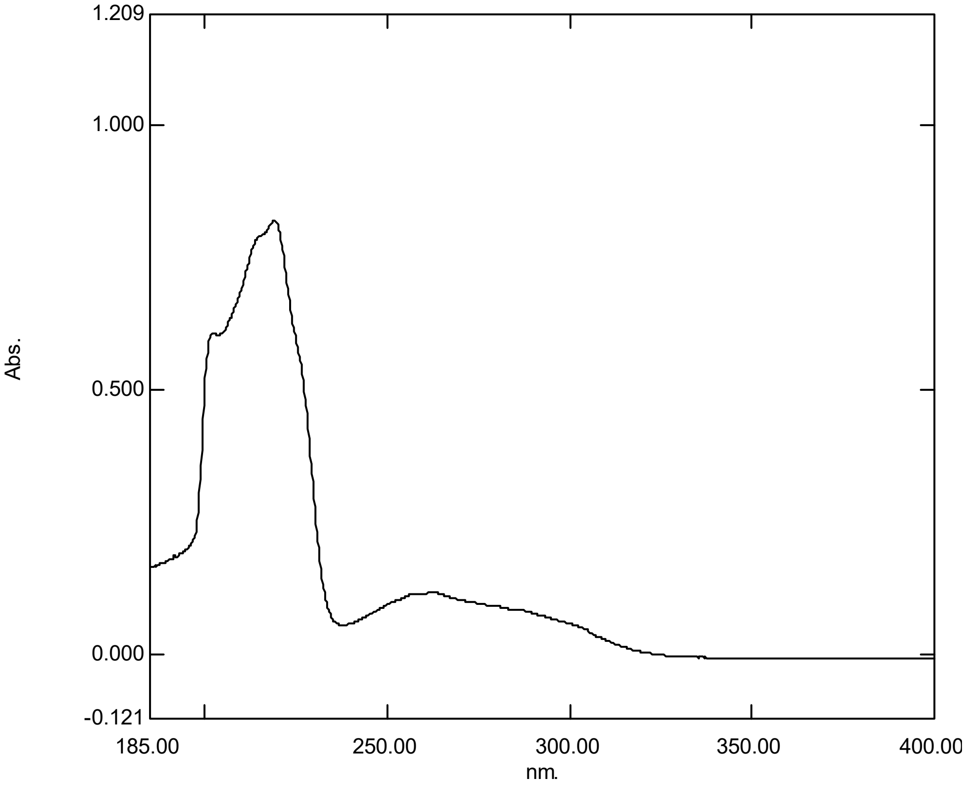

Figure S36. UV spectrum of compound **1**.

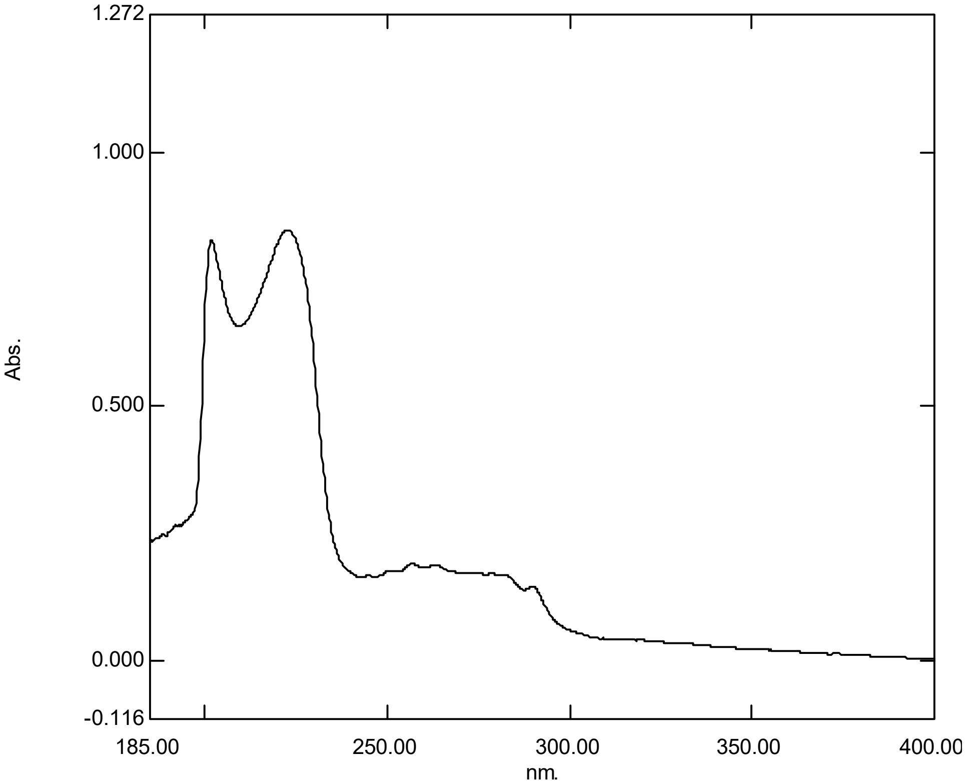

Figure S37. UV spectrum of compound **2**.

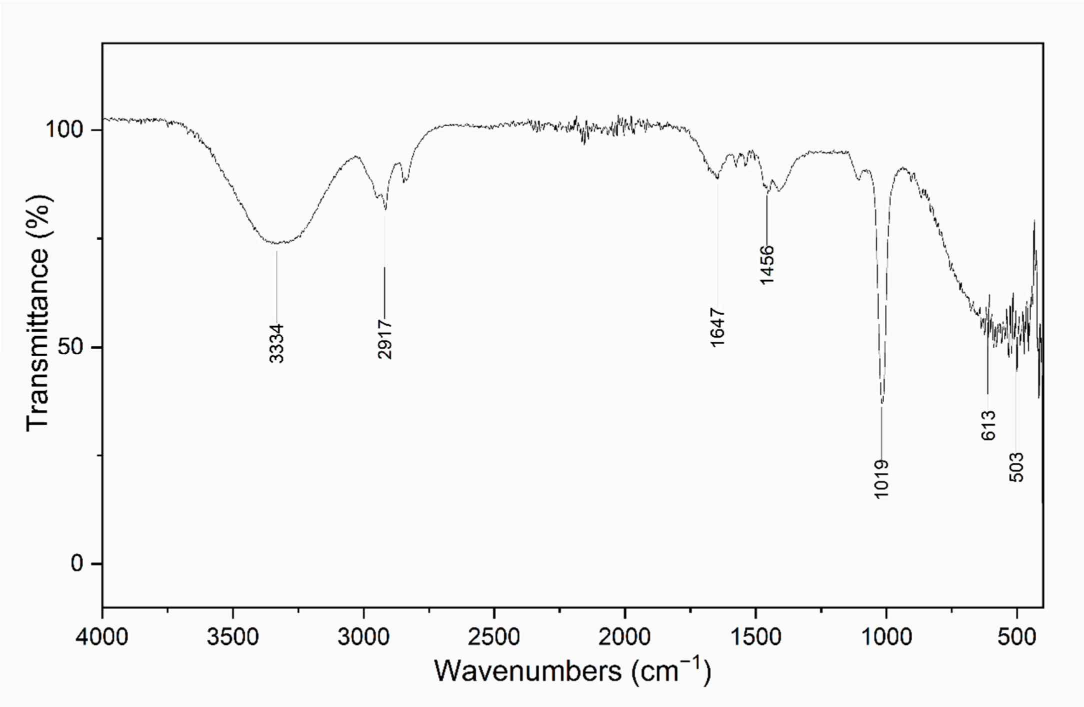

Figure S38. IR spectrum of compound **1**.

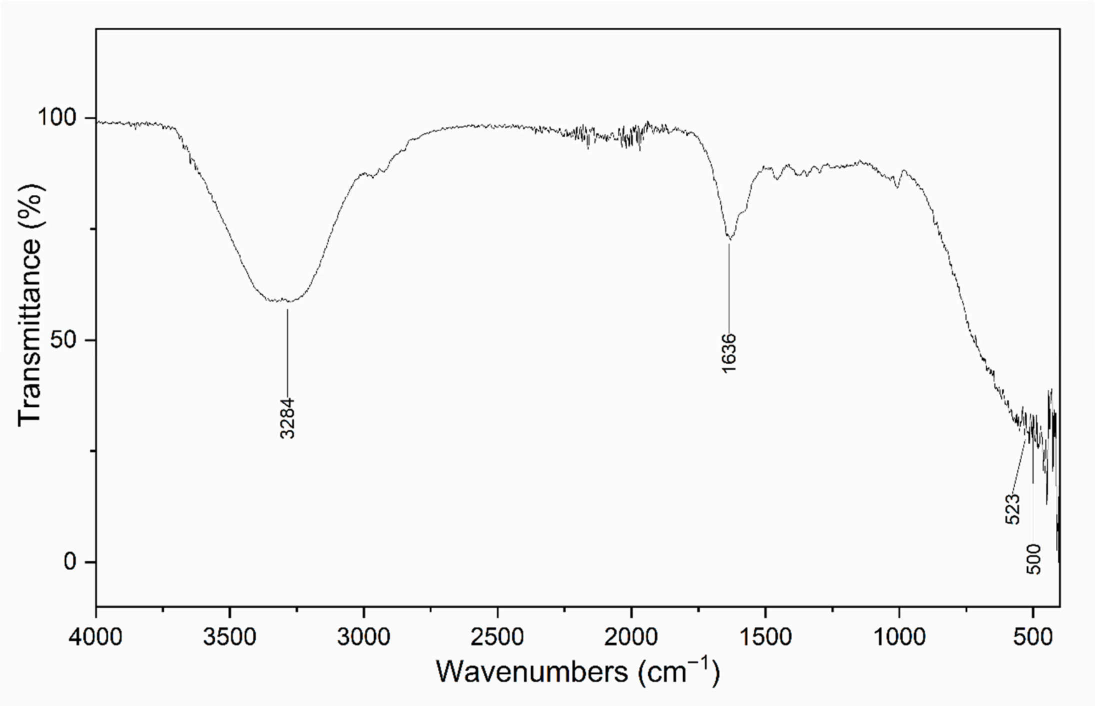

Figure S39. IR spectrum of compound **2**.

Data S3. Analysis of (+)-HR-ESI-MS/MS data of compound 1 and 5.

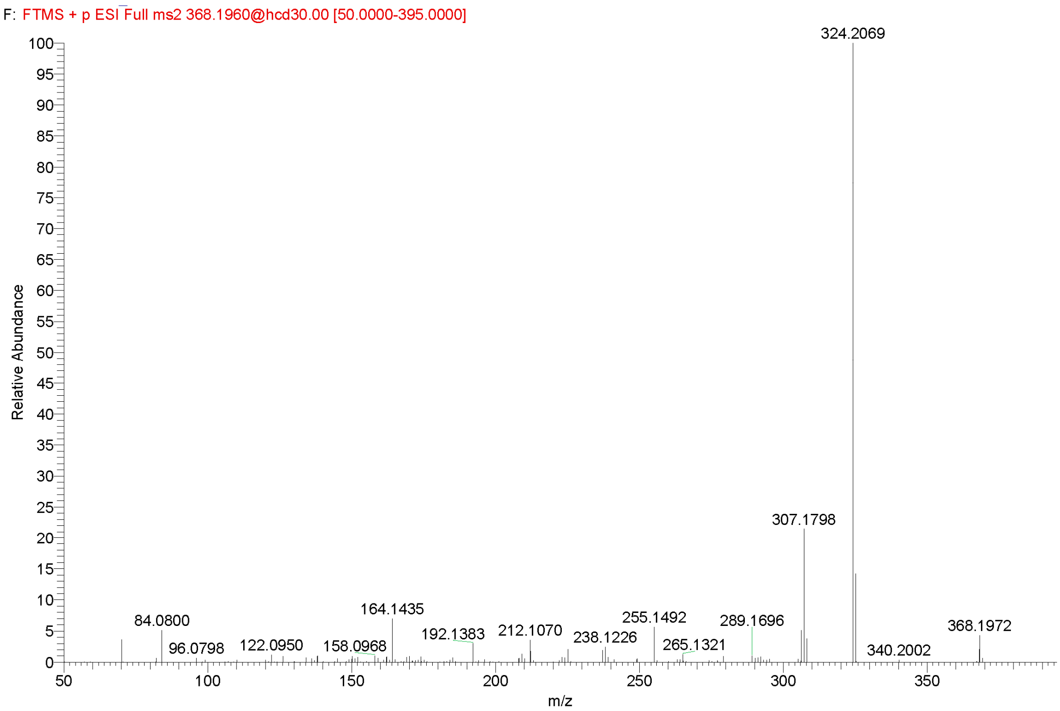

Figure S40. (+)-HR-ESI-MS/MS spectrum of **1**.

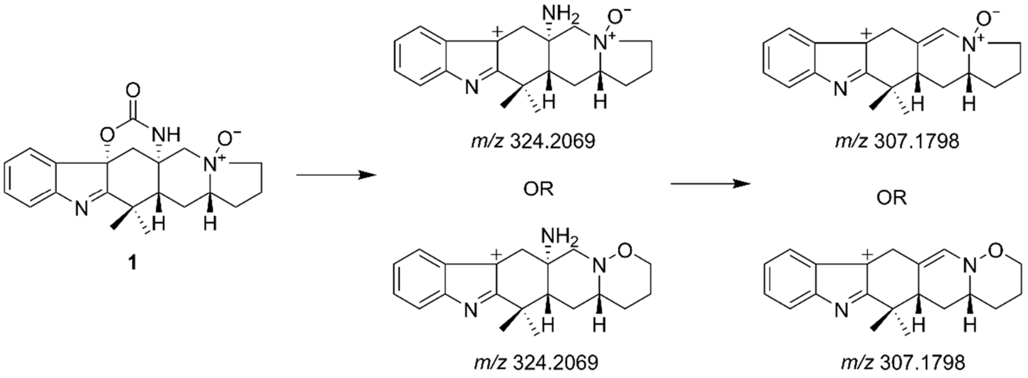

Scheme S1. A possible molecular cleavage process for **1**.

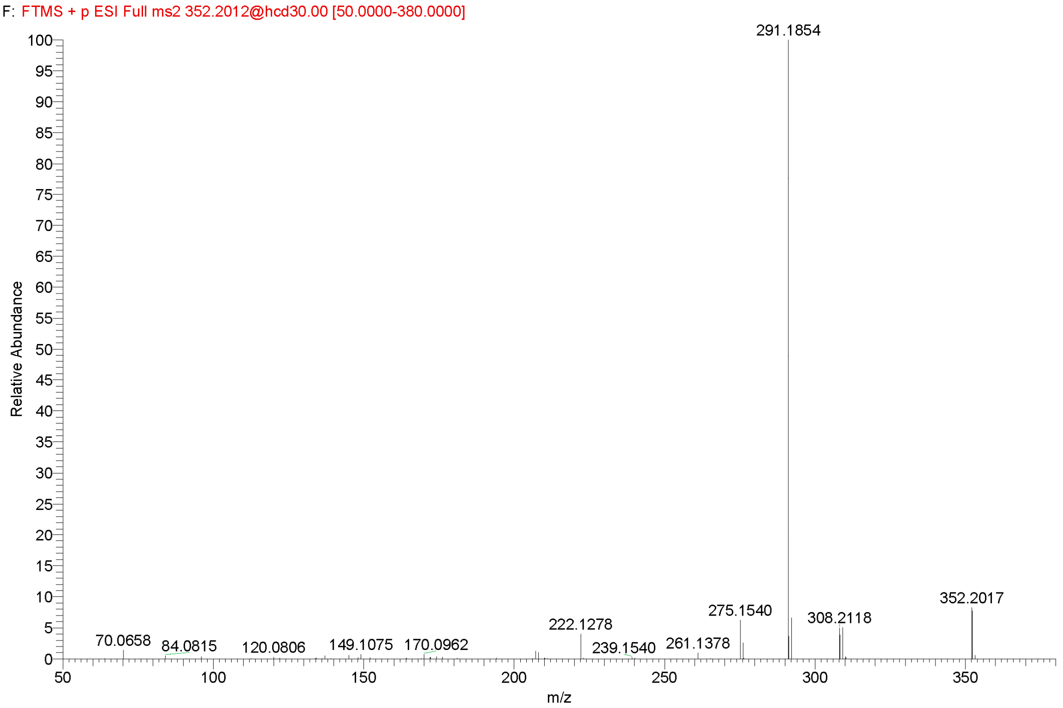

Figure S41. (+)-HR-ESI-MS/MS spectrum of **5**.

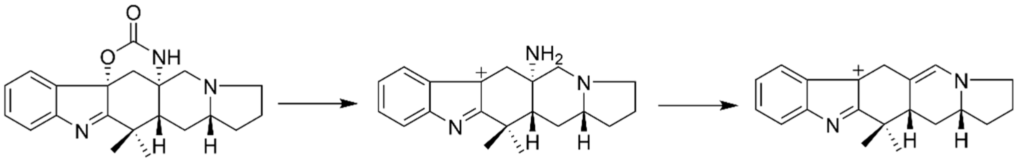

Scheme S2. A possible molecular cleavage process for **5**.

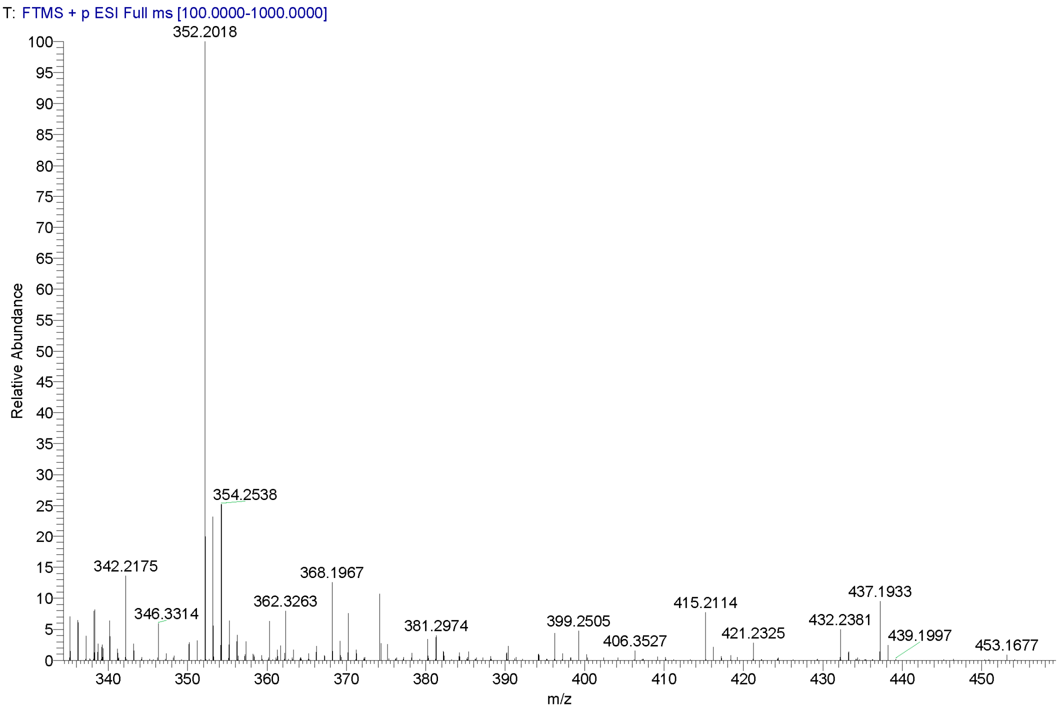

Figure S42. (+)-HR-ESI-MS spectrum of reaction product of **1**.

Data S4. Biological assay

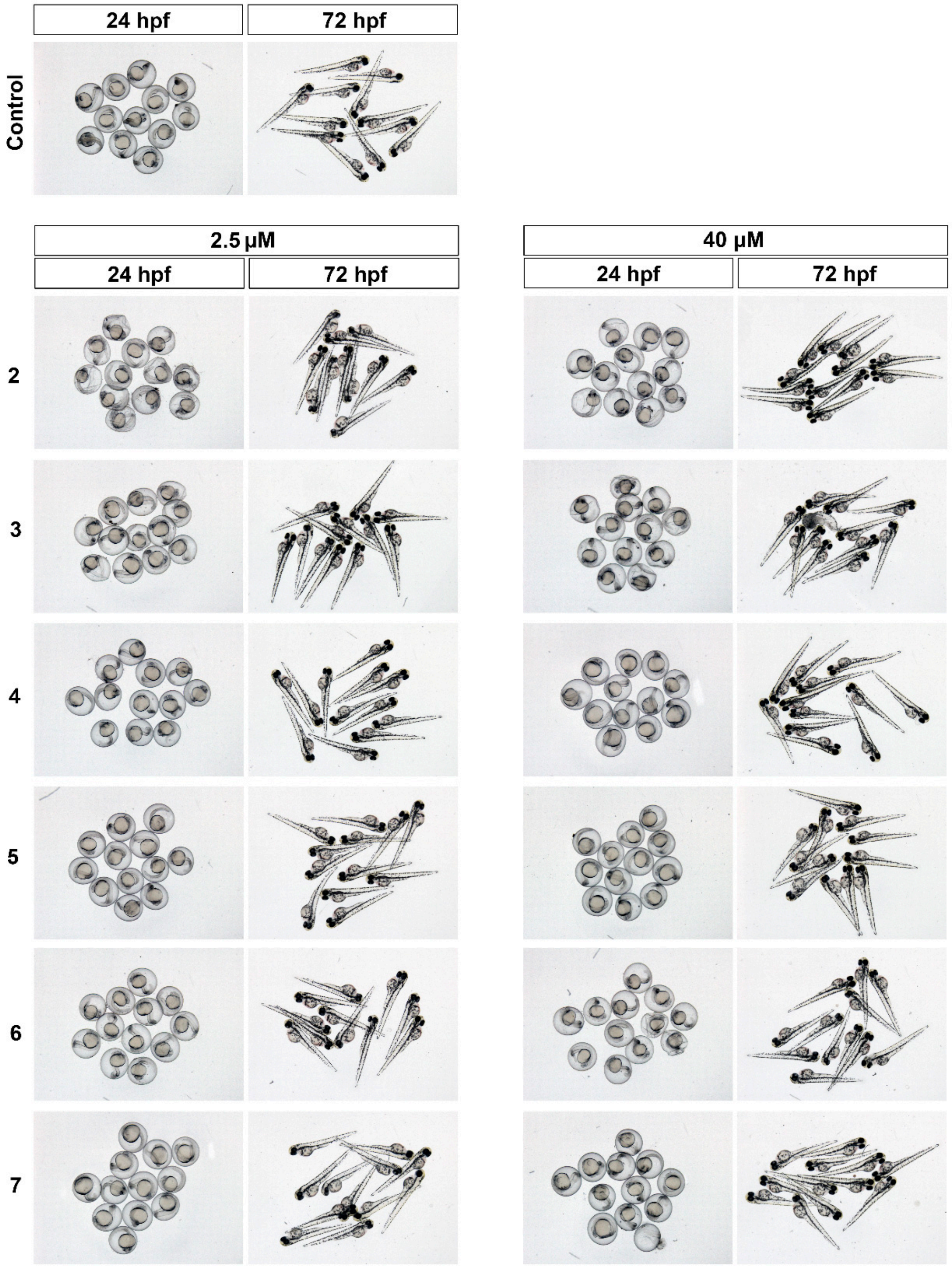

Figure S43. Morphology of 24 hpf embryos or 72 hpf zebrafish larvae treated with compounds **2-7**.

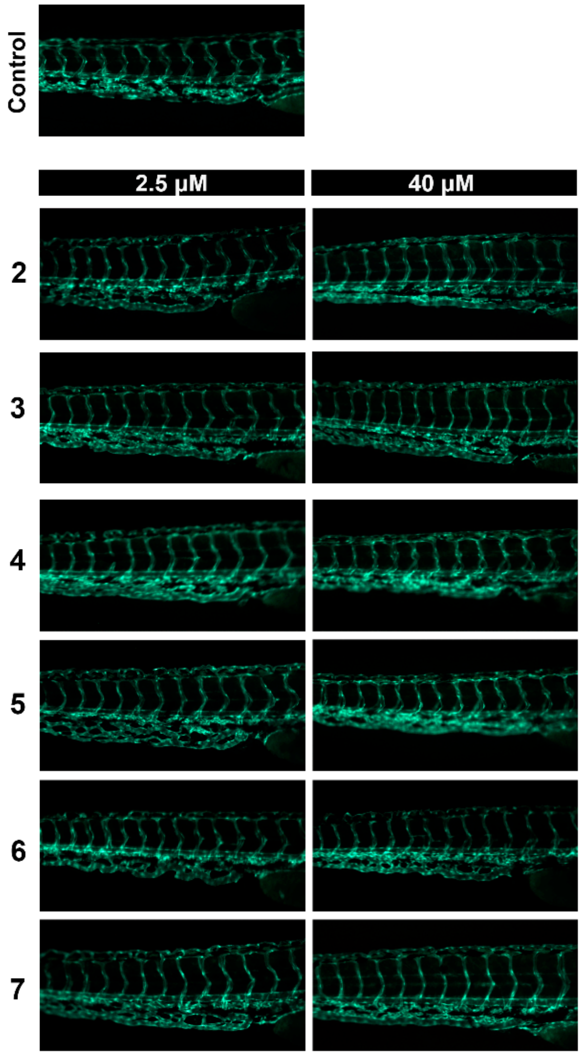

Figure S44. Blood vessels morphology of 6 dpf zebrafish larvae *Tg(flil:eGFP)* treated with compounds **2-7**.

Data S5. The BLAST search result of ITS sequence of fungus A-S-6.

Pallidocercospora crystallina strain ZJUM 2 18S ribosomal RNA gene, partial sequence; internal transcribed spacer 1, 5.8S ribosomal RNA gene, and internal transcribed spacer 2, complete sequence; and 28S ribosomal RNA gene, partial sequence.

Sequence ID: KP896014.1 Length: 556 Number of Matches: 1

Range 1: 2 to 509 GenBank Graphics ▼ Next Match ▲ P

Score	Expect	Identities	Gaps	Strand
929 bits(503)	0.0	508/510(99%)	2/510(0%)	Plus/Minus

```
Query  1    CCGAGGTCAACCTTGTGAAAGATTTAACGGCCGCGGCCACCCGACCTCCGAAGCGAATAG  60
            ||||||||||||||||||||||||||||||||||||||||||||||||||||||||||||
Sbjct  509  CCGAGGTCAACCTTGTGAAAGATTTAACGGCCGCGGCCACCCGACCTCCGAAGCGAATAG  450

Query  61   TTGCCACAACGCTTAGAGACGGACAGCTCAGCCGGAAGACTTTAAGGCGCGCGGAGCCGC  120
            ||||||||||||||||||||||||||||||||||||||||||||||||||||||||||||
Sbjct  449  TTGCCACAACGCTTAGAGACGGACAGCTCAGCCGGAAGACTTTAAGGCGCGCGGAGCCGC  390

Query  121  GACGCCCAATACCAAGCCAGGCTTGAGTGGTGAAATGACGCTCGAACAGGCATGCCCCCC  180
            ||||||||||||||||||||||||||||||||||||||||||||||||||||||||||||
Sbjct  389  GACGCCCAATACCAAGCCAGGCTTGAGTGGTGAAATGACGCTCGAACAGGCATGCCCCCC  330

Query  181  GGAATACCAGGGGGCGCAATGTGCGTTCAAAGATTCGATGATTCACTGAATTCTGCAATT  240
            ||||||||||||||||||||||||||||||||||||||||||||||||||||||||||||
Sbjct  329  GGAATACCAGGGGGCGCAATGTGCGTTCAAAGATTCGATGATTCACTGAATTCTGCAATT  270

Query  241  CACATTACTTATCGCATTTCGCTGCGTTCTTCATCGATGCTGGAACCAAGAGATCCGTTG  300
            ||||||||||||||||||||||||||||||||||||||||||||||||||||||||||||
Sbjct  269  CACATTACTTATCGCATTTCGCTGCGTTCTTCATCGATGCTGGAACCAAGAGATCCGTTG  210

Query  301  TTGAAAGTTTTGTTTAATTTACTTTAACTCCGACGCAATGATGCAGTGTTTAATGGCCTC  360
            ||||||||||||||||||||||||||||||||||||||||||||||||||||||||||||
Sbjct  209  TTGAAAGTTTTGTTTAATTTACTTTAACTCCGACGCAATGATGCAGTGTTTAATGGCCTC  150

Query  361  CGGGGGCGCCGCACCGCCGAAGCGGCAGGGTCGCCCCCGAAGCAACAAGTTTGGTTCACA  420
            ||||||||||||||||||||||||||||||||||||||||||||||||||||||||||||
Sbjct  149  CGGGGGCGCCGCACCGCCGAAGCGGCAGGGTCGCCCCCGAAGCAACAAGTTTGGTTCACA  90

Query  421  AAGGGTTGGAGGTCGGACCGAAGCCCTCACTCAGTAATGATCCCTCCGCAGGTTCACCTA  480
            ||||||||||||||||||||||||||||||||||||||||||||||||||||||||||||
Sbjct  89   AAGGGTTGGAGGTCGGACCGAAGCCCTCACTCAGTAATGATCCCTCCGCAGGTTCACCTA  30

Query  481  CGGAGACCTTGTTACGACTTTTTTACTTCC  510
            ||||||||||||||||||||||  ||||||
Sbjct  29   CGGAGACCTTGTTACGACTTTT--ACTTCC  2
```
